# Raman spectroscopy to unravel the magnetic properties of iron oxide nanocrystals for bio-related applications†

**DOI:** 10.1039/c9na00064j

**Published:** 2019-04-23

**Authors:** Martín Testa-Anta, Miguel A. Ramos-Docampo, Miguel Comesaña-Hermo, Beatriz Rivas-Murias, Verónica Salgueiriño

**Affiliations:** Departamento de Física Aplicada, Universidade de Vigo 36310 Vigo Spain vsalgue@uvigo.es; Université Paris Diderot, Sorbonne Paris Cité, ITODYS, UMR CNRS 7086 75013 Paris France

## Abstract

Iron oxide nanocrystals have become a versatile tool in biomedicine because of their low cytotoxicity while offering a wide range of tuneable magnetic properties that may be implemented in magnetic separation, drug and heat delivery and bioimaging. These capabilities rely on the unique magnetic features obtained when combining different iron oxide phases, so that an important portfolio of magnetic properties can be attained by the rational design of multicomponent nanocrystals. In this context, Raman spectroscopy is an invaluable and fast-performance tool to gain insight into the different phases forming part of the nanocrystals to be used, allowing correlation of the magnetic properties with the envisaged bio-related applications.

## Abstract 2

Raman spectroscopy is a very valuable and fast-performance tool to gain insight first into the different iron oxide phases present in nanoparticles, to correlate then the magnetic properties with potential bio-related applications.

## Graphical abstract

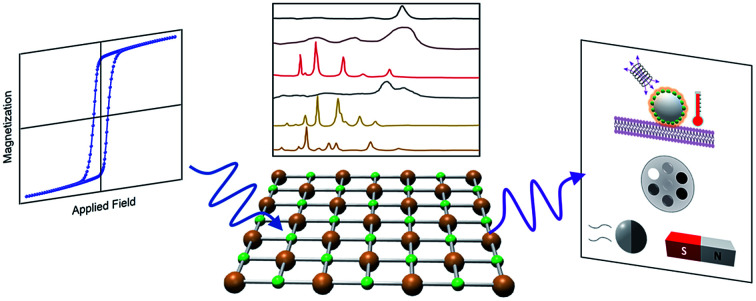

## 1. Introduction

Iron oxide nanocrystals are ubiquitous in the field of biomedicine due to their low cytotoxicity and the huge platform of magnetic properties that they display. In this regard, their size, morphology, crystallinity, oxidation state and surface functionalization are the main parameters dictating the final bio-related applications for which they are conceived (*i.e.* magnetic separation, drug delivery, hyperthermia and bioimaging).^1–5^ A more complex scenario is present in multicomponent iron oxide nanocrystals, where the relative distribution and physical interactions of the different phases have an enormous impact on the final magnetic properties of the material.^6,7^ Accordingly, the appearance of new magnetic features in iron oxide heterostructures opens the door to improved performances in these applications.

As the complexity of magnetic nanostructures increases, more sophisticated characterization tools are required to ascertain the intrinsic relation between the chemical composition and the magnetic features observed. This specificity is particularly relevant in the cases where chemical segregation or cation doping lead to the formation of complex interfaces between the different magnetic components.^8,9^ Along these lines, previous studies exemplify this novel trend in which the combination of complementary techniques and/or the use of state-of-the-art characterization tools allow for a more profound understanding of the chemical composition/distribution in complex magnetic materials. In a recent report, Garnero and coworkers have taken advantage of a multi-technique approach to understand the chemical ordering of FeCo nanoparticles. In this work the combined use of X-ray diffraction, Mössbauer spectroscopy and zero field ^59^Co ferromagnetic nuclear resonance (FNR) has allowed for the first-time observation of short-range B2 ordering in FeCo nanoparticles synthesized under mild conditions.^10^ In another example, high-resolution scanning transmission electron microscopy (HR-STEM) has been used to characterize the complex interface created in magnetite nanoparticles coated with Mn_3_O_4_.^11^ More precisely, electron energy-loss spectroscopy (EELS) measurements performed with atomic resolution permit a precise analysis of the oxidation state of the different chemical species present at the interface formed between both oxides. While these are just two examples of many advanced protocols developed to attain a deeper understanding of the structural properties of complex magnetic nanomaterials, we herein attempt to provide a comprehensive overview of current trends in the characterization of single- and multi-component iron oxide phases at the nanoscale using Raman spectroscopy. Namely, a direct correlation between the structural/chemical characteristics of the detected species and their magnetic properties will be established, paying special attention to the most relevant features for their implementation in bio-related applications.

Current reports addressing the characterization of iron oxide nanocrystals are based on multi-platform characterization, in which three are mainstream tools: while transmission electron microscopy (TEM) and X-ray diffraction (XRD) are used to investigate the morphological and structural characteristics, magnetometry (SQUID or VSM) is needed to understand the magnetic response of the material under study. That is, complementary techniques are used to characterize in detail the inner morphology and crystalline structure, as well as the magnetic behavior stemming from both of them.

Furthermore, many spectroscopic techniques can be also considered to broaden the range of information that can be obtained from complex magnetic composites. Among them, vibrational spectroscopic techniques such as IR and Raman can be particularly helpful. These techniques, based on the processes of absorption and inelastic scattering of light, respectively, can provide valuable information on the chemical structure, the identification of species through their spectral ‘fingerprints’, and even allow for a (semi-)quantitative analysis. Though both techniques are actually complementary, Raman spectroscopy has multiple advantages over IR absorption: it is a non-invasive and non-destructive technique (under controlled conditions); it works in a short timescale; it does not require sample preparation; and samples can be examined in the gaseous, liquid and solid form. Moreover, the most environmentally sensitive bands stemming from the presence of adsorbed water molecules are broad and weak, hence overcoming one of the main limitations of IR spectroscopy.

When dealing with crystalline structures such as transition metal oxide nanocrystals, rather than discrete molecules, the use of Raman spectroscopy is particularly convenient. Indeed, since most metal–oxygen lattice vibrations occur below 700–750 cm^−1^, this technique provides a greater selectivity due to fewer spectral overlaps. In addition, careful analysis of the Raman spectra can also provide a deeper insight into the crystal structure, which is sometimes not accessible by other vibrational spectroscopic techniques. For instance, Raman spectroscopy can be used to address surface oxidation frequently observed in many materials at the nanoscale (generally not detected by either IR or XRD).^12^ It is also an alternative and complementary technique to elucidate the doping and cation redistribution and inversion effects (often reported in spinel oxides),^13–15^ and to identify magnetite or maghemite (in general very difficult using XRD),^16^ and structural and phase transitions that may be induced as a consequence of the laser power.^17,18^ On top of that, and in line with the scope of this review, magnetic phenomena such as magnons (*i.e.* spin waves) and spin-phonon coupling have also been registered by Raman spectroscopy,^19,20^ and analogously, information related to electric properties or mechanical properties can be obtained from dielectric or ceramic nanocomposites, respectively.

Nevertheless, and in spite of the remarkable benefits that Raman spectroscopy entails for the structural elucidation of complex nanocrystals, a reduced number of studies have taken advantage of this technique.^13,16–18,21–23^ In the present work the relevance of Raman spectroscopy as an invaluable tool to obtain chemical and structural information on complex magnetic oxides will be discussed at length. For a better comprehension and interpretation, these results are preceded by an introductory section covering the fundamentals of this technique, paying special attention to crystalline iron oxides.

## 2. Basics of Raman spectroscopy

### 2.1 Basic principles

**Fig. 1:**
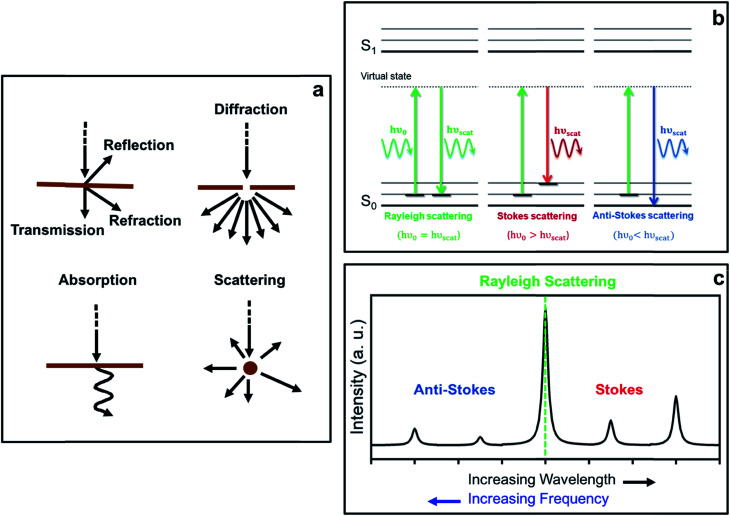
Diagrams showing (a) different processes resulting from light-matter interactions, (b) Rayleigh and Raman Stokes and anti-Stokes scattering processes from a microscopic point of view, and (c) a typical Raman spectrum displaying the relative intensities of the different scattering processes.

The interaction of light with matter can lead to several different processes (see schemes in Fig. 1a). In fact, when the energy of the incident light matches exactly the energy gap between the ground state and an excited state associated with the material, the incident photons may be absorbed, thereby promoting the system to that excited state. This constitutes the basis of absorption spectroscopic techniques, such as IR (infra-red) spectroscopy, where the loss of radiation of a particular energy is detected.

The incident photons may also interact with matter by distorting (polarizing) the electron cloud around the nuclei and, as a result, promote the system to a higher-energy state typically referred to as a ‘virtual state’, which is not a proper state of the system. Indeed, this ‘virtual state’ is no longer stable and the photons are readily re-radiated, giving rise to the phenomenon of light scattering. In this case, the photons do not require an energy which matches the difference between two states. Raman spectroscopy is based on this scattering process.

From a classical approach, light can be described as an oscillating electromagnetic field that is perpendicular to the wave propagation direction, forming a transverse wave.^24^ For light of frequency *ν*_0_, the magnitude of the oscillating electrical field is given by:

1 *E* = *E*_0_ cos(2π*ν*_0_*t*)

where *E*_0_ is the amplitude and *E* is the magnitude of the electrical field at time *t*.

Upon applying a field ***E***, an electric dipole moment ***μ*** that oscillates in phase with the electrical field will be induced in the system. This induced dipole moment is proportional to the applied field and can be calculated according to the following expression:

2 *μ* = *αE* = *αE*_0_ cos(2π*ν*_0_*t*)

where *α* represents the electrical polarizability of the system.

During a particular vibration k, the polarizability of the system will change as a result of nuclear displacement. Accordingly, the polarizability *α* can be expanded in a Taylor series in terms of the normal coordinates *q*_k_ of the nuclear displacements as follows:3
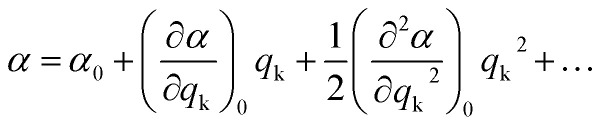
where *α*_0_ stands for the polarizability at the equilibrium position (*q*_k_ = 0).

For small vibrations, they can be regarded as harmonic oscillations, and thus the normal coordinates are given by:

4 *q*_k_ = *q*^0^_k_ cos(2π*ν*_k_*t*)

where *ν*_k_ and *q*^0^_k_ represent the frequency and amplitude of a specific normal mode k.

Approximating eqn (3) to the first order term and inserting the two previous equations into eqn (2), gives:5
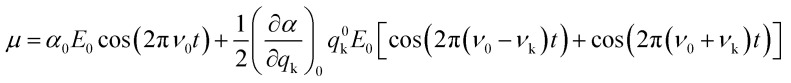


From eqn (5), it can be inferred that an electric dipole oscillating at a frequency *ν*_0_ will lead to photons of three different frequencies. The first term, known as Rayleigh scattering, is unshifted in frequency (*ν*_0_) and corresponds to an elastic dispersion of light, since after being excited to the ‘virtual state’ the system drops back to the same initial state. Conversely, the second and third terms represent an inelastic dispersion of light, in which the oscillating frequency differs from that of the incident light by either *ν*_0_ − *ν*_k_ (Stokes scattering) or *ν*_0_ + *ν*_k_ (anti-Stokes scattering). These two processes give rise to the phenomenon of Raman scattering, where a net energy transfer between the incident light and the system takes place. In the case of Stokes scattering the incident photons promote the system from the ground to a ‘virtual state’ and transfer some finite energy to the system, as the system drops down to a higher energy vibrational state in the ground state. Alternatively, in the case of anti-Stokes scattering, the system loses energy by departing from an excited vibrational state before going back to a lower energy vibrational state in the ground state (see schemes in Fig. 1b).

In a typical Raman experiment the intensity of light scattering is represented against the Raman shift, Δ*ν̄* (in wavenumbers), which is defined as the shift in frequency of the scattered radiation (*ν*_scat_) with respect to that of the incident light (*ν*_0_):6
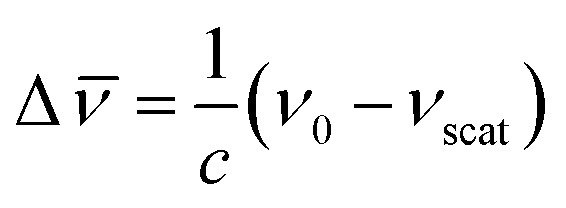


As mentioned before, the shift in frequency due to Raman scattering is determined by the spacing between the vibrational levels of the system, and hence, the Raman shift corresponding to Stokes and anti-Stokes scattering will be exactly the same but different in sign. This leads to a symmetrical spectrum (with respect to Rayleigh scattering) that displays (a) a null Raman shift for Rayleigh scattering, (b) a positive Raman shift for Stokes scattering, reflected on the lower frequency side of the spectrum, and (c) a negative Raman shift for anti-Stokes scattering, reflected on the higher frequency region of the spectrum (see Fig. 1c).

The intensity of Raman scattering is proportional to the fourth power of the incident light frequency (*ν*_0_), and from a classical point of view, it can be calculated according to the following expression:^25^7
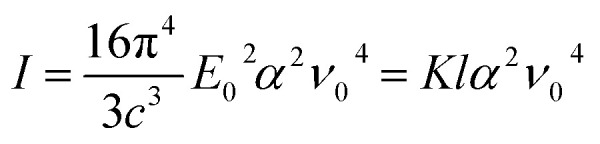
where *K* is a constant that depends on the speed of light *c* and *l* is the laser power (which is found to be proportional to *E*_0_^2^). According to eqn (7), the intensity of Raman scattering can be maximized by increasing the laser power and/or setting the frequency of the incident light to shorter wavelengths. However, a balance concerning these two parameters must be established so as to avoid sample degradation and secondary processes such as fluorescence (see description of the Raman equipment in the ESI†). In any case, it is important to highlight that inelastic scattering is inherently a weak phenomenon, since it is typically overshadowed by the far more prominent Rayleigh scattering. Indeed, only one of about 10^6^ to 10^8^ photons undergoes Raman scattering.

One more aspect to take into account is the fact that owing to the different departure vibrational levels involved, the intensity of the Stokes and anti-Stokes components will be different. The ratio of the intensities of Stokes and anti-Stokes scattering is dependent on the initial state population. Bearing in mind that in an anti-Stokes process the system departs from an excited vibrational state, statistical mechanics predicts a lower population and consequently a lower intensity for anti-Stokes scattering. Actually, this ratio of intensities can be determined from the Boltzmann vibrational distribution function:^26^8
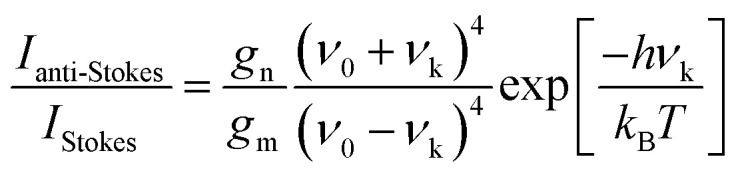
where *g*_m_ and *g*_n_ are the degeneracy of the departure vibrational levels m and n, *ν*_0_ is the frequency of the incident light, *ν*_k_ is the frequency of the vibration (which matches exactly the energy gap between the vibrational levels m and n), *k*_B_ is the Boltzmann constant and *T* is the temperature of the system. Thus, in line with eqn (8), the intensity of anti-Stokes scattering will increase along with the temperature, since higher energy vibrational states will become more populated.

As mentioned before, the different bands displayed in a Raman spectrum provide information about the internal vibrational structure (*i.e.* molecular vibrations in the case of discrete molecules and phonons/lattice vibrations in the case of crystals) of the system. Nonetheless, not all vibrations will be Raman active. In fact, as indicated by eqn (5) the two components of Raman scattering will vanish if the differential term is equal to zero, a condition that is governed by the symmetry of the system. This gives rise to the selection rule of Raman spectroscopy, which states that, in order to be Raman active, a vibration k must involve a change in the polarizability of the system, that is:9
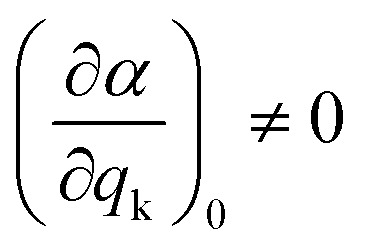


Since the polarizability can be thought of as the volume of the system, intense Raman scattering will occur from symmetric vibrations, as they imply a rather large change in polarizability compared to asymmetric vibrations. This is in contrast to IR spectroscopy, where the selection rules allow for transitions that imply a change in the dipole moment, which typically corresponds to asymmetric vibrations. Moreover, in the case of centrosymmetric systems, the rule of mutual exclusion is verified, that is, Raman active vibrations are IR inactive and vice versa, making Raman and IR spectroscopy complementary techniques.

Though the previous mathematical treatment can be useful when dealing with highly symmetrical systems (*i.e.* perfectly isotropic media), in non-isotropic media the response to an electric field will vary depending on the direction considered. Sometimes, the application of an electric field ***E*** in one direction may even lead to an induced dipole moment ***μ*** in a different direction. Therefore, in order to account for these anisotropic systems, the scalar treatment of the polarizability should be replaced by a second rank tensor, typically known as the polarizability tensor *α*:10
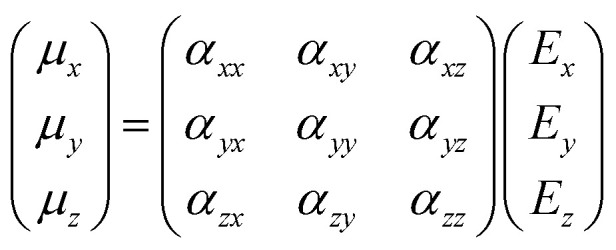
where the first subscript refers to the direction of polarization of the system and the second one to the direction of polarization of the incident light. Since the polarizability tensor is a symmetric matrix (*α*_*ij*_ = *α*_*ji*_), there are eventually six independent components, which renders the Raman selection rules less stringent compared to other vibrational spectroscopic techniques such as IR. Therefore, the selection rule of Raman spectroscopy should be reformulated in such a way that, if any of the components of the polarizability tensor changes during a particular vibration k, then that vibration is Raman active:11
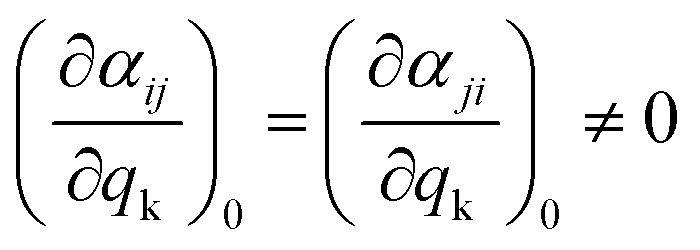


### 2.2 Group theory

When it comes to applying Raman spectroscopy to crystalline samples with the aim of identifying their crystalline structure, it becomes mandatory to foresee the associated vibrational modes. In this regard, the study of the vibrational modes assigned to a particular crystal lattice can be addressed by a factor analysis based on group theory. This is indeed a very convenient and powerful method, since not only does it predict the number of vibrational modes that will be Raman or IR-active, but also allows us to carry out an unequivocal indexation according to their specific local symmetry. Given the main topic of this review article, focusing on iron oxides, with magnetite (Fe_3_O_4_) being ubiquitous in light of its very remarkable magnetic properties, we will use the identification of the vibrational modes assigned to the magnetite spinel crystalline structure as an example. A simpler and more detailed approach for an AB_4_ discrete molecule is included in the ESI.†

The spinel structure has the cubic *Fd*3̄*m* (no. 227) space group or, as usually stated from a spectroscopic point of view, it displays an *O*_h_^7^ symmetry (under the Schönflies notation). This symbol comprises a set of different symmetry species and operations that can be summarized in the form of a character table on the basis of its point group (see Table 1 (ref. 27)).

**Table 1:** Character table for the *O*_h_ point group displaying the different symmetry species and symmetry operations for the spinel structure

*O*_h_ (*m*3̄*m*)	*E*	8*C*_3_	6*C*_2_	6*C*_4_	3*C*_2_	*i*	6*S*_4_	8*S*_6_	3*σ*_h_	6*σ*_d_		(*h* = 48)
A_1g_	1	1	1	1	1	1	1	1	1	1		*x* ^2^ + *y*^2^ + *z*^2^
A_2g_	1	1	−1	−1	1	1	−1	1	1	−1		
E_g_	2	−1	0	0	2	2	0	−1	2	0		(2*z*^2^ − *x*^2^ − *y*^2^, *x*^2^ − *y*^2^)
T_1g_	3	0	−1	1	−1	3	1	0	−1	−1	(*R*_*x*_, *R*_*y*_, *R*_*z*_)	
T_2g_	3	0	1	−1	−1	3	−1	0	−1	1		(*xy*,*xz*,*yz*)
A_1u_	1	1	1	1	1	−1	−1	−1	−1	−1		
A_2u_	1	1	−1	−1	1	−1	1	−1	−1	1		
E_u_	2	−1	0	0	2	−2	0	1	−2	0		
T_1u_	3	0	−1	1	−1	−3	−1	0	1	1	(*x*, *y*, *z*)	
T_2u_	3	0	1	−1	−1	−3	1	0	1	−1		

The first row in Table 1 displays the number and type of symmetry operations present in the point group, whereas the first column lists the irreducible representations, that is, the symmetry species comprising the group, which are denoted by their corresponding Mulliken symbol. The correlations between the symmetry operations and the irreducible representations are given by a series of characters, *χ*_*v*_(*R*), which simply indicate the effect of the symmetry operation *R* upon a vector (or a set of vectors) *v*. Considering a particular basis *v* (*e.g.* the *x*, *y* and *z* axes of each atom in the unit cell), a collection of characters of all the operations in the group gives rise to a representation *Γ*. Though the resultant representation depends on the basis *v*, any representation can be eventually reduced to a linear combination of irreducible representations. Accordingly, these are referred to as reducible representations and their corresponding characters can be determined by means of the following expression:^28^

12 *χ*(*R*) = *ω*(*R*)(±1 + 2 cos *θ*)

where *χ*(*R*) is the character of the reducible representation for the symmetry operation *R*, *ω*(*R*) is the number of atoms that remain invariant under the same operation and *θ* is the rotation angle.

The spinel unit cell is made up of eight face-centered cubic (fcc) cells (*i.e.*, *Z* = 8), offering 56 atoms per unit cell (32 oxygen atoms, 8 A atoms and 16 B atoms). Since only two octants along the main body diagonal of the spinel unit cell are certainly different, the calculation of the reducible representation can be addressed more easily by making use of a primitive cell (*i.e.*, the smallest rhombohedral Bravais cell), which comprises two fcc unit cells and 14 atoms. Consequently, taking into account eqn (12), the reducible representations corresponding to both a full and a primitive spinel unit cell can be readily calculated (see Table 2).

**Table 2:** Reducible representations of a full and primitive spinel unit cell

	*E*	8*C*_3_	6*C*_2_	6*C*_4_	3*C*_2_	*i*	6*S*_4_	8*S*_6_	3*σ*_h_	6*σ*_d_
*ω*	56	20	8	0	8	16	8	20	0	32
*Γ*_full_	168	0	−8	0	−8	−48	−8	0	0	32
*Γ*_primitive_	42	0	−2	0	−2	−12	−2	0	0	8

As stated before, these reducible representations can be expressed as a linear combination of the irreducible representations:^29^13
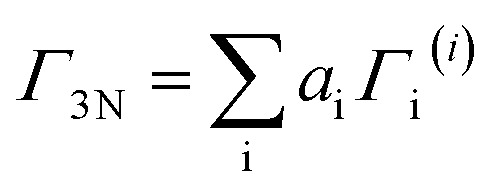
where *Γ*_3N_ is the reducible representation, *Γ*_i_^(*i*)^ is each irreducible representation contained in the point group, and *a*_i_ is an integer coefficient given by:14
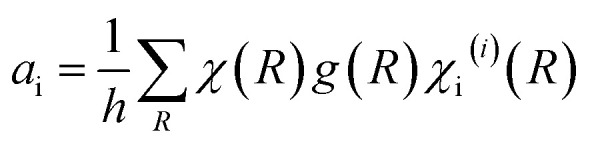
where *χ*(*R*) and *χ*_i_^(*i*)^(*R*) are the characters of the reducible and the *i*-th irreducible representation for the symmetry operation *R* respectively, *g*(*R*) is the number of symmetry operations *R* of the same class, and *h* is the order of the group.

Recalling the characters of the reducible and irreducible representations for the spinel structure (summarized in Tables 1 and 2), and bearing in mind that *h* is equal to 48 in the *O*_h_ point group, the previous reducible representations can be reduced as follows:

15 *Γ*_3N_ = A_1g_ + E_g_ + T_1g_ + 3T_2g_ + 2A_2u_ + 2E_u_ + 5T_1u_ + 2T_2u_

The number of vibrational modes in a particular crystal lattice is related to the number of times each irreducible representation is contained within the reducible representation. Therefore, it becomes necessary to work out which of the previous normal modes are actually purely optical vibrational modes. These optical vibrational modes can be thought of as lattice waves occurring due to an out-of-phase displacement of the atoms within the crystal lattice, and consequently they can be excited through conventional spectroscopic techniques, since they can interact with externally applied electromagnetic fields such as light. Contrarily, the acoustic modes occur due to an in-phase displacement of the atoms within the crystal lattice and cannot be detected with these techniques, as they propagate with the speed of sound (of a much lower frequency).

As shown in eqn (15), factor group analysis predicts 42 normal modes, though only 39 are truly vibrational normal modes. Indeed, as it can be inferred from the *O*_h_ character table, three of them (in the T_1u_ mode) represent pure translations and appear as acoustic modes involved in the propagation of sound waves through the crystal.^28^ By neglecting this acoustic T_1u_ mode, we can eventually obtain the irreducible representations of the optical phonon modes of the spinel structure:

16 *Γ*_vib_ = A_1g_ + E_g_ + T_1g_ + 3T_2g_ + 2A_2u_ + 2E_u_ + 4T_1u_ + 2T_2u_

Among them only some will be Raman and/or IR active. Accordingly, and as already mentioned, for a particular vibration to be Raman active a change in polarizability must occur, whereas a change in the dipolar moment is required to be IR active. Taking these selection rules into account, five vibrational modes are expected to be Raman active (A_1g_ + E_g_ + 3T_2g_, see schemes of these vibrational modes in Fig. S10, in the ESI†) and four IR active (4T_1u_), rendering the remaining vibrational modes inactive. Moreover, given that the space group *Fd*3̄*m* is centrosymmetric, the rule of mutual exclusion is verified, and hence Raman active modes are IR inactive and vice versa.

## 3. Iron oxides

**Fig. 2:**
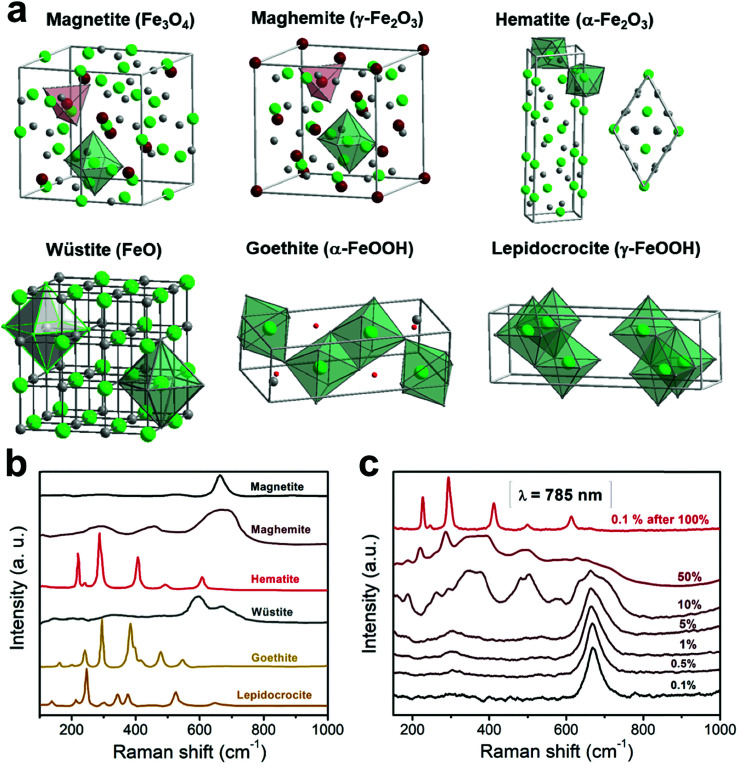
Crystalline structures (a) and the representative Raman spectra (b) of the most typical iron oxides and oxyhydroxides present in nanoparticles for bio-related applications (data taken and adapted from ref. 16, 31 and 42). Raman spectra showing the heat-induced transition from magnetite to maghemite and finally to hematite (c).

There exist sixteen iron compounds, including oxides, hydroxides and oxyhydroxides.^30^ Among them, the most common and important ones when designing magnetic nanoparticles for bio-related applications are magnetite, maghemite, hematite, wüstite, goethite and lepidocrocite, with the last two mainly being secondary phases. These materials exhibit distinct Raman signatures stemming from their different crystalline structures (see schemes in Fig. 2a), hence making Raman spectroscopy a very appropriate technique to discern their presence in the system under study, which is very much required given their commanding role in the final magnetic properties displayed.

### 3.1 Magnetite (Fe_3_O_4_)

This material exhibits a black color and the presence of iron ions in divalent and trivalent states. It crystallizes in an inverse cubic spinel structure (*Fd*3̄*m* space group, number 227), with an *a* cell parameter of 0.8396 nm. The spinel structure contains 8 formula units with a total of 56 atoms per unit cell, though only 14 atoms are necessary to construct the simplest primitive cell (*i.e.* two molecular AB_2_O_4_ units). This structure can be described in terms of cubic close-packing (fcc, face-centered cubic lattice) of oxygen anions with 1/8 of the tetrahedral and 1/2 of the octahedral holes occupied, out of the 64 tetrahedral and 32 octahedral sites available in the spinel structure. It becomes particularly interesting to differentiate between the normal and inverse spinel structure. While the normal configuration has the divalent cations at the tetrahedral positions and the trivalent cations at the octahedral positions (for example ZnFe_2_O_4_ or CdFe_2_O_4_), in the inverse spinel the divalent cations are located at the octahedral sites whereas the trivalent cations are equally distributed between the tetrahedral and octahedral sites (besides the magnetite, for example CoFe_2_O_4_). Furthermore, spinels with a partial inverse structure (for example MgFe_2_O_4_ and MnFe_2_O_4_) can be also found.

Applying group theory to the spinel structure (as described in Section 2.2), five vibrational modes are expected to be Raman active: A_1g_ + E_g_ + 3T_2g_. Reported values in the literature for these vibrations are 193 (T_2g_(1)), 306 (E_g_), 450–490 (T_2g_(2)), 538 (T_2g_(3)) and 668 cm^−1^ (A_1g_) for the magnetite case.^21^ A typical Raman spectrum corresponding to magnetite is shown in Fig. 2b (in black), with a characteristic intense peak at ∼660–670 cm^−1^ (A_1g_) and two less intense peaks at ∼300 (E_g_) and ∼530 cm^−1^ (T_2g_(3)).^21,31^ The weaker T_2g_(2) and T_2g_(1) modes, on the other hand, are not always documented in the magnetite Raman spectrum, particularly if registering the vibration modes at room temperature, or from nanometer-sized samples.

Though there is some controversy in the assignment, most authors claim that the presence of the A_1g_ band is related to the symmetric stretching of oxygen atoms (‘breathing motion’) in the tetrahedral FeO_4_ group, along the 〈111〉 direction,^32^ whereas the lower frequency modes are associated with different motions in the tetrahedral unit.^21,33^ In this regard, based on polarization measurements, Shebanova *et al.* assigned the vibrational modes E_g_ and T_2g_(2) to the symmetric and asymmetric bending of oxygen with respect to iron in the tetrahedral void, respectively. The remaining two Raman modes were reported to involve the motion of both oxygen and iron cations at the tetrahedral sites:^21^ the T_2g_(3) mode through an asymmetric stretching of iron and oxygen, and the T_2g_(1) mode through the complete translation of the FeO_4_ unit within the spinel unit cell (schemes of the vibrational modes are included in Fig. S10 in the ESI†).

In general, when considering an inverse spinel compound displaying two different types of cations at the octahedral sites, the corresponding Raman spectrum shows a splitting of the A_1g_ mode. As pointed out by Laguna *et al.*,^34^ this A_1g_ splitting can be explained according to a scenario where the tetrahedral units are not completely isolated, but surrounded by three other octahedral units. As a consequence, this mode involves the stretching of oxygen atoms in the tetrahedral void together with the deformation of three metal–oxygen bonds at the octahedral sites.

Magnetite displays a characteristic crystalline transition from a cubic to a monoclinic lattice, known as Verwey transition (*T*_v_ = 119 K), which also involves changes in its magnetic, electrical (metal-insulator transition) and thermal properties. From a magnetic point of view, this material is ferrimagnetic with a Curie temperature *T*_C_ = 860 K and a saturation magnetization value of 92 A m^2^ kg^−1^ at 300 K (in bulk). In terms of thermal stability, magnetite transforms firstly into maghemite and finally into hematite upon heating in air. This transition can be induced and studied by varying the laser power in Raman experiments, as a consequence of an energy transfer which locally heats the crystalline structure (Fig. 2c).

### 3.2 Maghemite (γ-Fe_2_O_3_)

This material has a red-brown color and consists of a fully oxidized iron oxide in which all iron cations are in a trivalent state. It also has a spinel crystalline structure and accordingly, the structure is a fcc oxygen array with 1/8 of the tetrahedral sites and 5/12 of the octahedral sites occupied, displaying the following formula: (Fe^3+^)[Fe_5/3_^3+^□_1/3_]O_4_, where () and [] denote the tetrahedral and octahedral sites respectively, and □ denotes the iron vacancies. The distribution of these vacancies in the crystalline structure can be ordered or disordered, and three possible crystal symmetries have been proposed.^30,35,36^ In a random distribution of these vacancies, the material crystallizes in the same cubic system (*Fd*3̄*m*) as the magnetite. In a similar distribution of vacancies as in the case of the lithium cation in LiFe_5_O_8_, the material shows a cubic space group *P*4_3_32 (or its enantiomorph *P*4_1_32) with a cell parameter of *a* = 0.8347 nm. Finally, in an ordered distribution it displays a tetragonal symmetry (space group *P*4_1_2_1_2 or its enantiomorph *P*4_3_2_1_2) and unit cell parameters *a* = 0.8347 nm and *c* = 2.501 nm. These different distributions of the cation vacancies usually stem from the preparation method used to attain this material, which transitions to hematite if heated above 400 °C (as shown in Fig. 2c, heating the sample with increasing laser power).

In the maghemite material, while for the cubic space group *P*4_3_32, the expected Raman vibrational modes are 6A_1_ + 14E + 20T_2_, in the case of a random distribution of the vacancies, group theory predicts the same number of vibrational modes as for magnetite. However, the more intense A_1_ peak appears shifted towards higher wavenumbers, around 700 cm^−1^.^16,37^ Additionally, two broad bands centered at ∼350 (T_1_) and ∼500 cm^−1^ (E), with completely different relative intensities in comparison to magnetite (Fig. 2a, in red-brown), are usually displayed.^37^

From a magnetic point of view, maghemite shows a ferromagnetic behavior with a Curie temperature *T*_C_ ∼ 985 K and a saturation magnetization value of ∼74 A m^2^ kg^−1^ at room temperature.

### 3.3 Hematite (α-Fe_2_O_3_)

Hematite is the most common iron oxide in nature and its color is blood-red. This material crystallizes in a corundum structure (α-Al_2_O_3_), which is based on a hcp (hexagonal close-packed) oxygen packing, with the iron cations occupying two-thirds of the octahedral sites. The space group is *R*3̄*c* in the rhombohedral system and the cell parameters are *a*_rh_ = 0.252 nm and *α* = 55.3° (for the hexagonal setting *a* = 0.5034 nm and *c* = 1.375 nm).

Applying group theory to the point group of hematite, seven vibrational modes are expected to be Raman active: 2A_1g_ + 5E_g_. The Raman spectrum for this material is shown in Fig. 2a (in red), where the most intense peak is located at ∼290 cm^−1^ (E_g_(2) + E_g_(3)), along with other features at 225 (A_1g_(1)), 247 (E_g_(1)), 412 (E_g_(4)), 498 (A_1g_(2)) and 613 cm^−1^ (E_g_(5)).^37,38^

This material is extremely stable and shows a canted antiferromagnetic behavior (*i.e.*, weakly ferromagnetic) between its Morin transition temperature (∼260 K) and its Néel temperature (*T*_N_ ∼ 960 K).^39^ Below the Morin transition temperature, the material is in an antiferromagnetic state.^40^

### 3.4 Wüstite (FeO)

This iron compound contains only divalent iron cations and its color is black. The crystal lattice is a rock-salt structure, based on a fcc (face-centered cubic) metal ion arrangement which crystallizes in the *Fm*3̄*m* space group (number 225). The unit cell parameter is *a* = 0.4302 nm. Despite the fact that group theory predicts wüstite to be a very weak Raman scatterer without proper Raman active modes, one mode at around 595 cm^−1^ has been reported for this phase in the literature and is related to an inelastic second harmonic light scattering process.^31^ Alternatively, other authors have reported very similar spectra to the ones from magnetite with the most intense peak located at 652 cm^−1^.^38^

From a magnetic point of view, this material is a type II antiferromagnetic material made up of alternating ferromagnetic [111] planes, with a Néel temperature *T*_N_ ∼ 203–211 K.

### 3.5 Goethite (α-FeOOH)

In the powder form the color of goethite is yellow-brown, rendering it an important pigment in industry. Its structure is based on hexagonal close-packing of anions which crystallizes in an orthorhombic system (*Pnma* space group, number 62). The unit cell parameters are *a* = 0.9956 nm, *b* = 0.30215 nm and *c* = 0.4608 nm.

Applying group theory to this structure, 24 vibrational modes are expected to be Raman active: 8A_g_ + 4B_1g_ + 8B_2g_ + 4B_3g_. The Raman spectrum (shown in Fig. 2b) includes two strong peaks located at 299 and 385 cm^−1^, besides other less intense peaks at 244, 480, 548 and 681 cm^−1^.^31^

This material is antiferromagnetic with a Néel temperature of 460 K.^39^

### 3.6 Lepidocrocite (γ-FeOOH)

This material exhibits an orange color and a layered crystal structure (isostructural with boehmite, γ-AlOOH), based on cubic close-packing (ccp) of anions (O^2−^/OH^−^) stacked along the [150] direction, with the iron cations located at the octahedral interstices. This material crystallizes in an orthorhombic system (*Bbmm* space group) with *a* = 1.2520 nm, *b* = 0.3873 and *c* = 0.3071 nm as cell parameters. *Bbmm* is a different setting compared to the standard *Cmcm* (number 63).

The application of group theory to this structure predicts nine Raman active modes: 3A_g_ + 3B_1g_ + 3B_3g_ (considering the same position of hydrogen and one of the oxygen atoms). Though there is less consistency in the literature in comparison to other iron compounds, the presence of a sharp and intense peak at 250 cm^−1^ in the Raman spectrum is generally agreed to be indicative of the presence of lepidocrocite. Additional features at 301, 348, 379, 528 and 650 cm^−1^ have also been reported for this structure,^31,41^ as shown in Fig. 2b (in orange). Below 70 K, the material is antiferromagnetic.

## 4. Bio-related applications using multicomponent iron oxide-based nanocrystals

The combination and distribution of some of the iron oxide magnetic phases described above within nanoparticles stem from the mechanism behind the designed synthetic route, which usually includes the attachment of ligands to the nanoparticle surface, for improving its colloidal stability. The method can therefore take advantage of different chemical reactions and/or phase transitions, eventually offering multicomponent iron oxide-based nanocrystals. Raman spectroscopy will register the vibrations stemming from the crystalline lattices and the vibrations from the molecules attached to the surface. Whereas most metal–oxygen lattice vibrations occur below 750 cm^−1^, the main vibrations of organic molecules take place above 1000 cm^−1^, and therefore, do not interfere with each other.^43–45^ This implies an arrangement of magnetic phases that will delineate the magnetic behavior. For instance, the presence of magnetic materials with different magnetic orders can establish exchange coupling between two phases, contributing to the effective magnetic anisotropy. This characteristic, along with shape anisotropy and magnetocrystalline anisotropy of the nanocrystals synthesized, determines a preferred magnetization direction within the material, and consequently, the final bio-related application. Accordingly, we will briefly detail the basics of the three main bio-related applications for which magnetic nanoparticles are required, namely magnetic separation, heat delivery and magnetic resonance imaging (MRI).

### 4.1 Magnetic separation

**Fig. 3:**
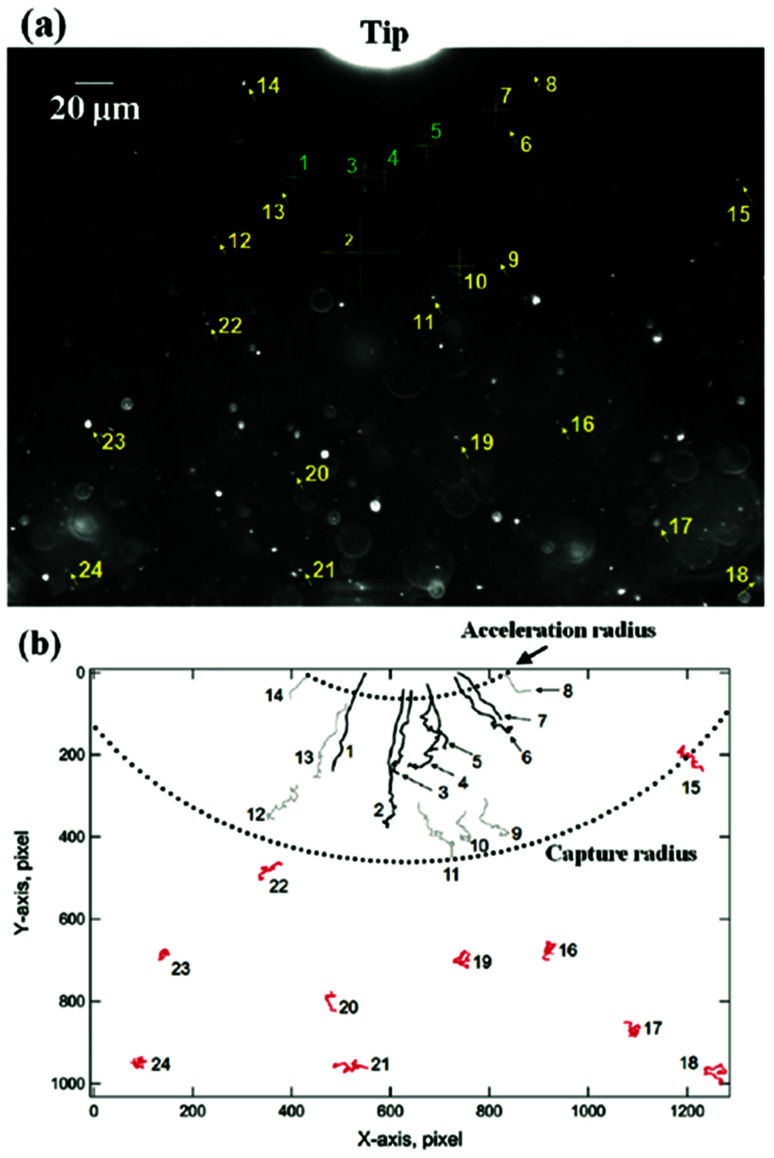
Dark field optical micrograph showing the initial position of the nanoparticles relative to a reference position (tip) (a), and their trajectories after undergoing diffusion (in red) or magnetophoresis (without (grey) and with acceleration (black)) in the direction of the magnetic field gradient (b). This figure has been reproduced from ref. 53 with permission from the American Chemical Society.

Magnetic separation considers the optimization of manipulation of nanoparticles at a distance using a magnetic field.^46^ For example, magnetically guided propulsion of nanostructures,^47^ magnetically targeted drug delivery^48–50^ or cell separation to enrich or deplete cells of interest from a heterogeneous cell population,^51,52^ take place *via* a fluid-based magnetic separation device. These phenomena require a magnetic field gradient to exert a force (see Fig. 3). Consequently, the optimization of specific magnetic parameters depending on the magnetic phases present in the nanoparticles or nanostructures employed should be taken into consideration.^23^ Otherwise, the use of large magnetic field gradients (for example up to 3000 T m^−1^ or 3 × 10^−3^ T per micrometer (ref. 53)) would be required.

In this context, for magnetic separation to be carried out the nanoparticles are required to respond magnetically, so that they can be guided to the right location. To attain such magnetic manipulation in solution, we have to take into account the mentioned magnetic force stemming from a magnetic field gradient (a uniform magnetic field would induce a torque or a moment of force on the magnetic object and would rotate it but not displace it). In order to obtain an effective magnetic manipulation, this magnetic force should overcome the hydrodynamic drag force due to the flowing solution.^6^ In this situation, spherical nanoparticles will reach a velocity relative to that of the carrier fluid defined by:17
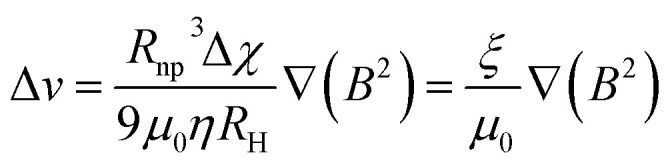
which depends on the magnetic field gradient (∇*B*^2^), the viscosity of the medium (*η*), the particle radius (*R*_np_), the hydrodynamic radius (*R*_H_), and the magnetic susceptibility of the magnetic nanoparticles relative to those of the carrier fluid (Δ*χ*). The velocity acquired by the magnetically manipulated nanoparticles is therefore linearly dependent on the ‘magnetophoretic mobility’ of the particle (*ξ*), which summarizes the size and magnetic characteristics of the nanoparticles and the fluid medium employed (eqn (18)).18
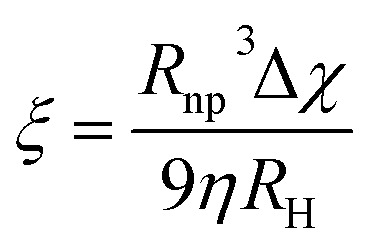


According to this ‘magnetophoretic mobility’ *ξ*, it is clear that for magnetic separation applications, nanoparticles of soft magnetic materials (large magnetic susceptibility at low fields) in their superparamagnetic state are mandatory, along with a relatively large size. However, magnetic nanoparticles with large sizes are generally not superparamagnetic but ferri- or ferromagnetic (below their Curie temperature), with large values of coercivity stemming from large total magnetic moments. Such a scenario would promote strong dipolar interactions and consequently, aggregation.

In view of the above discussed details, engineering magnetic nanoparticles with the right characteristics for magnetic separation is a subject of major importance. Along these lines, nanostructures based on clusters of nanoparticles, or nanostructures with a core–shell morphology are preferred. In the latter case two scenarios are possible: the use of a non-magnetic or diamagnetic material on which the magnetic nanoparticles are embedded or the use of a combination of two different magnetic phases (*i.e.* ferri- or ferro- and antiferromagnetic materials) to direct the final magnetic behavior. Attending first to magnetic nanoparticles grouped in clusters, these aggregates display collective properties depending on the composition, size, shape and interactions between the individual nanoparticles forming them. In this manner, the most important characteristics stem from the collectivity itself, that is, the large total magnetic moment stemming from a sum of the magnetic moment of superparamagnetic nanoparticles,^13,54,55^ and a large total size, consequently favoring large magnetophoretic mobility. This last feature has proven to be very convenient for recycling and reuse in sensing, catalysis or drug delivery applications.^56–59^

For the second case, that is, nanostructures with a core–shell morphology and with a non-magnetic or diamagnetic material on which the magnetic nanoparticles are embedded or deposited,^7,47,60,61^ the strategy works the following way: while a large core (of polystyrene or silica spheres) helps to increase the total size, the deposition of a large number of very small superparamagnetic nanoparticles increases the total magnetic moment, and as a result, these nanostructures display a rather large magnetophoretic mobility.

The third strategy works the same way, since while trying to increase the total size of a magnetic nanostructure, given its influence on the magnetophoretic mobility, the superparamagnetic behavior must be ensured to avoid aggregation. This can be attained by combining a ferri- or ferromagnetic material and an antiferromagnetic material. The most characteristic case would consist of nanoparticles of metallic iron (ferromagnetic) which naturally oxidize (reaching an antiferromagnetic phase), or the typical wüstite–magnetite (antiferromagnetic–ferrimagnetic phases) combination, though most of these cases correspond to rather small nanoparticles.^62–67^ Nevertheless, the same arrangement of iron oxides but considering larger nanostructures will have a tremendous impact on the magnetophoretic mobility.^23^ In order to shed light on the role of Raman spectroscopy to unravel the magnetic properties of these nanostructures, considering the different magnetic compounds forming them in these detailed strategies, here we have discussed a few examples.

**Fig. 4:**
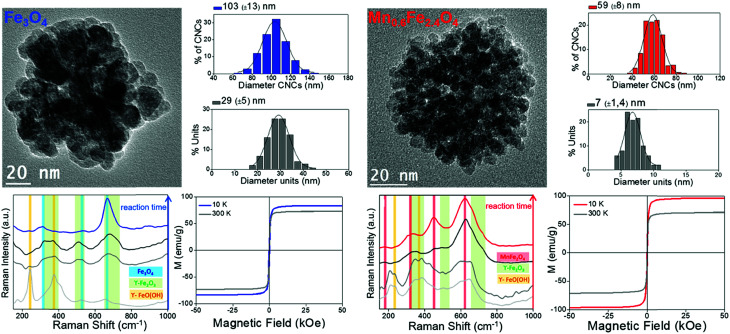
Summary of the characterization of clusters of magnetite (left, blue) and Mn-doped magnetite (right, red) with TEM images, size distribution analysis (of the clusters and the nanocrystals forming them), evolution of the Raman spectra during the formation of the clusters using a 785 nm excitation wavelength and hysteresis loops.

For the first strategy, that is, magnetic nanoparticles grouped in clusters, we can illustrate the benefits of these nanostructures for magnetic manipulation using two examples reported recently.^13^ Clusters of magnetic nanoparticles of two different spinel ferrites (Fe_3_O_4_ and Mn_0.6_Fe_2.4_O_4_) were prepared following a solvothermal method, by which both the total average size of the clusters and the average size of the nanoparticles forming them can be tuned. Fig. 4 includes the description of the two types of clusters, with representative TEM images of the magnetite and manganese ferrite clusters with average sizes of 103 ± 13 nm and 59 ± 8 nm, respectively. Moreover, the size of the individual units forming the clusters differs from one sample to another, again being bigger than those of the magnetite sample (29 nm *vs.* 7 nm). Therefore, the three parameters that change between both samples are the nature of the magnetic material, the total size of the clusters and that of the individual units forming them. The magnetic material defines or delimits the value of magnetic susceptibility, which is key in terms of the magnetophoretic mobility. Furthermore, the total size of the clusters is very important as well for the magnetophoretic mobility, being linearly dependent. When it comes to the average size of the magnetic nanoparticles forming the clusters, this parameter is crucial to have the final clusters in a superparamagnetic state so that their aggregation can be avoided.

Raman spectroscopy plays its role when it comes to knowing the combination of oxides present in these clusters, which induces certain phase transitions.^13^ Actually, the reaction mechanism behind the formation of the final material (Fe_3_O_4_ and Mn_0.6_Fe_2.4_O_4_) in the clusters was demonstrated using Raman spectroscopy (using a 785 nm excitation wavelength) in combination with X-ray diffraction. In both cases, the final clusters have evolved through a series of poorly crystalline intermediates (lepidocrocite phase) into the more stable spinel structure of maghemite, with subsequent reduction to magnetite or to the non-stoichiometric manganese ferrite, respectively, as confirmed with the magnetic characterization during the final stage. These results are a good example in which Raman spectroscopy (alone and/or in combination with other techniques) becomes a powerful tool to study the nature of poorly crystalline intermediates that are frequently obtained in the colloidal synthesis of nanoparticles.

**Fig. 5:**
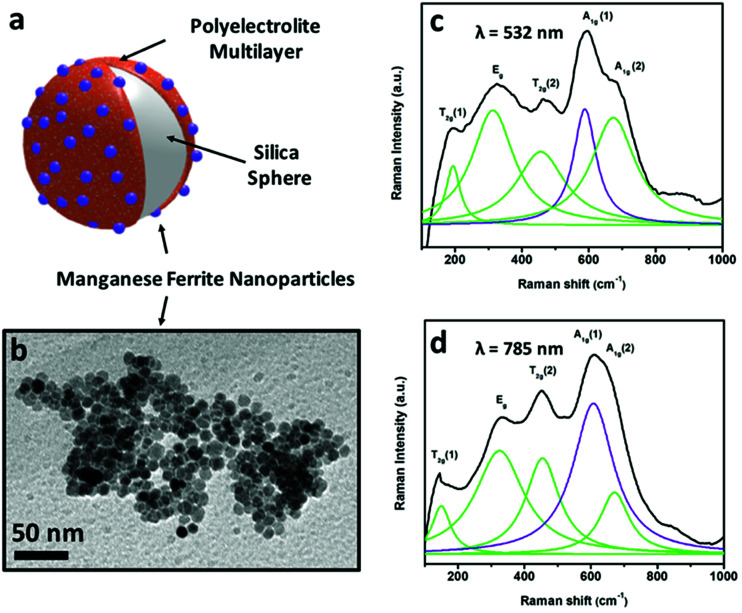
Scheme (a) of a substrate to form part of a self-propelled swimmer, magnetically functionalized with MnFe_2_O_4_ nanoparticles shown in the TEM image (b). Raman spectra of these manganese ferrite nanocrystals recorded using two excitation wavelengths (532 nm (c) and 785 nm (d)).

The second strategy by which the magnetophoretic mobility can be increased has also some illustrating examples. For instance, nanoswimmers that can self-propel because of different reactions can be magnetically guided, given the fact that manganese ferrite nanoparticles were included in the nanostructures, either with one, two or three layers of these nanoparticles adsorbed onto a silica substrate that holds the whole nanoswimmer together (see scheme in Fig. 5a). With this type of strategy (a non-magnetic or diamagnetic material on which the magnetic nanoparticles are adsorbed) the large substrate employed ensures size contribution. Moreover, it is particularly important to use a magnetic material with large susceptibility, aiming at a large value of magnetophoretic mobility. This characteristic can be accomplished with manganese ferrite nanoparticles whose structure and hence, magnetic properties can be unraveled using Raman spectroscopy.

According to group theory, manganese ferrite presents the same five Raman active modes as other spinels. Four main bands (fitted to Lorentzian curves in green) can be observed in the Raman spectra included in Fig. 5 (using excitation wavelengths of *λ* = 532 nm (c) and *λ* = 785 nm (d)), which correspond to the vibrational modes T_2g_ (1), E_g_, T_2g_ (2), A_1g_ (1) and A_1g_ (2), respectively. Nevertheless, registering the Raman modes using two different excitation wavelengths permits a more detailed analysis. Both the 532 nm- and 785 nm-excitation wavelength spectra can resolve the A_1g_ band, which when fitted to Lorentzian curves, split into two different modes,^68^ centered at 588–608 and ∼670 cm^−1^ (in violet and green), pointing out the presence of two different cations (Mn^2+^ and Fe^3+^) in the tetrahedral positions of the spinel structure, attending to the fact that manganese ferrite can have a partially inverse spinel formula. The presence of manganese cations in the tetrahedral positions induces an increased value of magnetization compared to magnetite (inverse spinel), and consequently, a larger value of susceptibility at low fields.

**Fig. 6:**
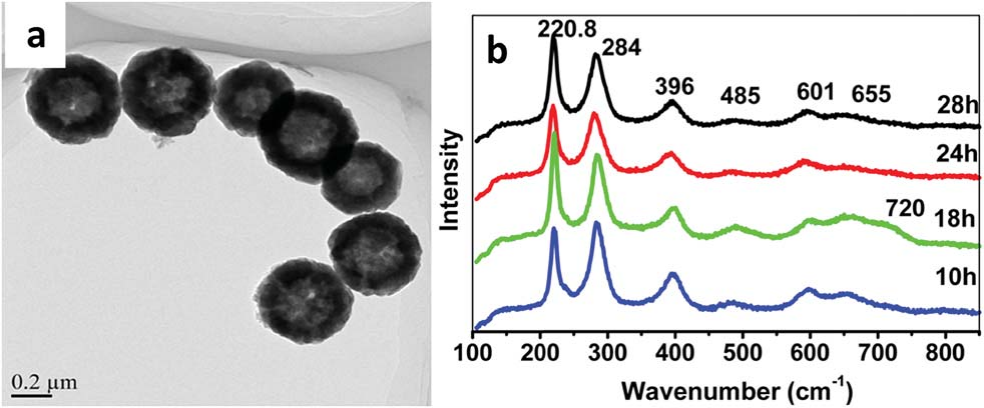
TEM image and Raman spectra of mesocrystalline Zn-doped Fe_3_O_4_ hollow submicrospheres. This figure has been adapted from ref. 69 with permission from the American Chemical Society.

This type of strategy with a non-magnetic or diamagnetic material and ferrimagnetic nanoparticles was also smartly achieved by synthesizing magnetic recyclable mesocrystalline Zn-doped Fe_3_O_4_ hollow submicrospheres (see a TEM image in Fig. 6a), thereby ensuring both the large size and large magnetic susceptibility required for magnetic manipulation of these nanostructures after a catalytic cycle.^69^ For that, magnetite nanocrystals were first assembled in clusters and further coated with a Zn-rich shell, such that, while promoting a Kirkendall effect for the hollow structure, the Zn^2+^ ions gradually diffused into the Fe_3_O_4_ nanocrystals. Raman spectroscopy using a 633 nm excitation wavelength at different powers revealed the presence of different oxides (mainly Fe_3_O_4_ and/or ZnFe_2_O_4_ when using 20% power, or both Fe_3_O_4_ and/or ZnFe_2_O_4_ and jointly α-Fe_2_O_3_, and γ-Fe_2_O_3_) because of an induced phase transition at the surface of the nanostructures, when using 40% power (as shown in Fig. 6b).

The third strategy proposed, a combination of iron oxides (wüstite–magnetite) in relatively large nanostructures, has also been studied using Raman spectroscopy, with magnetite (Fe_3_O_4_) as the major phase. However, using a 785 nm laser source having a higher penetration depth and a small shoulder at the prominent A_1g_ mode of the spinel structure indicated the additional presence of wüstite and residual traces of hematite.^23^ Another aspect to point out when performing the Raman study is the absence of changes after recording different subsequent spectra. The absence of any partial transition during this characterization indicates that the nanocrystals had reached a stable composition, despite the energy input of this procedure.

### 4.2 Heat delivery

Magnetic hyperthermia or heat delivery is based on the intrinsic capacity of magnetic particles to deliver heat by mechanisms stemming from hysteresis losses, or Néel and Brownian (if the material is dispersed in a liquid phase) relaxations.^70^ For nanoparticles in the superparamagnetic state and exposed to an alternating magnetic field, the effective relaxation time is given by Shliomis' equation:^71^19
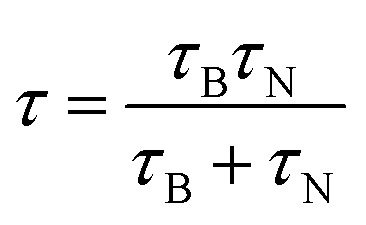
where *τ*_N_ is the magnetic (spin–spin) relaxation, also known as Néel relaxation, and *τ*_B_ is the viscous (particle–solvent) or Brownian relaxation, both defined by the expressions:20
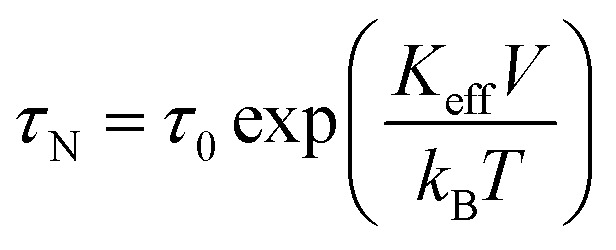
21
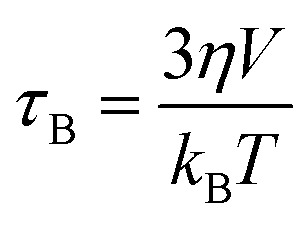
where *τ*_0_ is the Larmor precession time (∼10^−9^ to 10^−13^ s), *K*_eff_ is the effective magnetic anisotropy, *V* is the magnetic volume of the nanoparticle, and *η* is the viscosity of the medium. Recently, an equipotential diagram for ruling out the dominant relaxation mechanism was reported.^72^ In general terms, while high viscous media hinder Brownian relaxation, thus blocking the rotation of the particles to deliver energy, low viscous media permit the manifestation of both mechanisms.

The heat capacity of a randomly oriented nanoparticle of a ferri- or ferromagnetic material (below the Curie temperature) or a superparamagnetic nanoparticle can be tabulated in the form of its power density loss (*P*),^73^ given by eqn (22) and (23), respectively:22
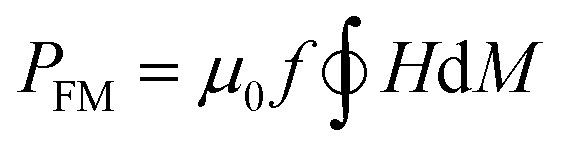
23
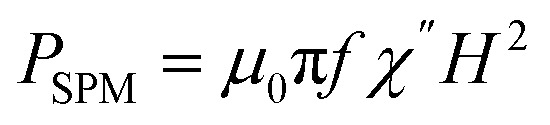
where *f* is the nominal frequency of the alternating magnetic field of amplitude *H* and *M* is the magnetization of the material, or where *f* is the nominal frequency of the alternating magnetic field, and *χ*′′ is the imaginary part of the volumetric magnetic susceptibility of the material, respectively. The field amplitude and frequency must be kept below the physiological limit when considering clinical applications (*H*_0_*f* < 5 × 10^9^ A m^−1^ s^−1^).^70^

According to the expressions, while in the first case the power density loss is directly proportional to the enclosed area of the hysteresis loop, for superparamagnetic nanoparticles it scales up in a quadratic fashion along with the magnetic field, and linearly with the imaginary part (*χ*′′) of the volumetric magnetic susceptibility:^74^24
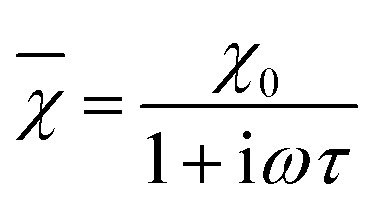
with real (*χ*′) and imaginary (*χ*′′) components (dependent on *ω* = 2π*f*):25a
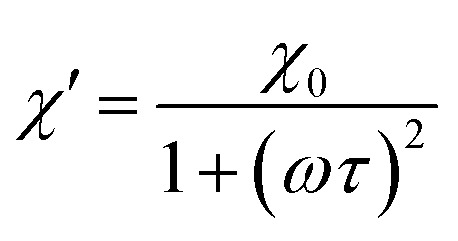
25b
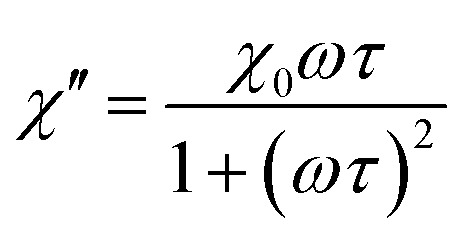


Taking into account the dependence of the susceptibility on frequency (eqn (24), (25a) and (25b)), the relation between the imaginary part of the susceptibility *χ*′′, the relaxation time *τ* and the power density *P* can be deduced.

In a biological context, the heat capacity of the nanoparticles is defined by a parameter known as the specific absorption rate (SAR) (or the specific loss power, SLP), which is related to the absorbed power per mass of the magnetic material, in units of watt per gram:26
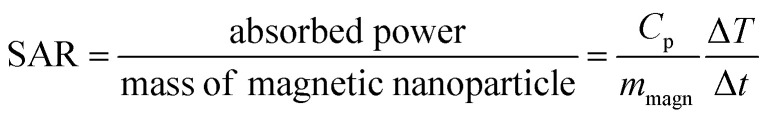
where *C*_p_ is the heat capacity of the fluid (usually water), *m*_magn_ is the mass referred to the magnetic material, and Δ*T*/Δ*t* is the heating rate.

**Fig. 7:**
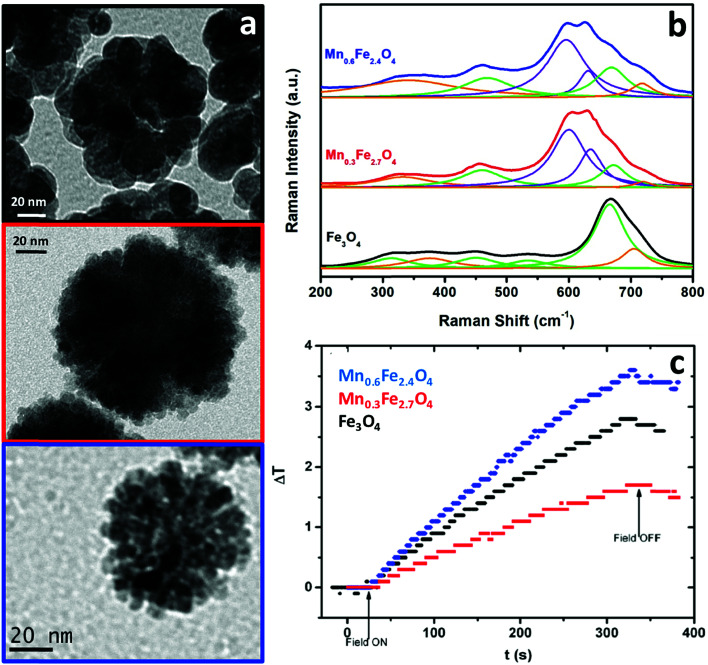
TEM images (a), Raman spectra (b) and temperature profile during the application of an alternating magnetic field (c) of clusters of Fe_3_O_4_, Mn_0.3_Fe_2.7_O_4_ and Mn_0.6_Fe_2.4_O_4_ nanoparticles.

Due to these features, and analogous to the previous case of magnetic separation, a thorough analysis of the magnetic nanoparticles put into play for the heat delivery is therefore required in order to clearly identify the iron oxide phases that denote the behavior in terms of the hysteresis loops or the magnetic susceptibility. However, there are very few cases using Raman spectroscopy for this aim. We have reported similar Mn-doped magnetite clusters of nanoparticles as those described in the previous section, as potential candidates for magnetic hyperthermia applications (Fig. 7a).^54^ In order to clarify the degree of substitution of Fe^2+^ by Mn^2+^ and possible oxidation transitions favored during the synthesis (Fig. 7b), Raman spectroscopy was therefore used. Appropriately, we were able to identify the characteristic A_1g_ vibrational mode of magnetite (black spectrum), centered at 670 cm^−1^ (fitted to a Lorentzian curve in green). In addition, maghemite was also identified in this pristine sample with no Mn doping, as a broadening of the A_1g_ vibrational mode with a characteristic shoulder at 700 cm^−1^ was observed (this band is fitted in orange in the figure). On increasing the Mn doping (red and blue spectra in Fig. 7b), this A_1g_ vibrational mode appears shifted towards 600 cm^−1^ (fitted in violet) when Mn cations were introduced into the nanostructure. This shift in the A_1g_ vibrational mode of the spinel stems from the new cation distribution, with Fe and Mn cations in the tetrahedral positions. There is indeed an additional A_1g_ vibrational mode at 640 cm^−1^ (fitted in violet) which is due to fact that some of the manganese cations of the clusters of Mn-doped magnetite nanoparticles have been oxidized.^75^

While magnetite and maghemite are both active phases for heat delivery, the hyperthermia properties of the nanoclusters were modified with manganese doping. For the hyperthermia experiments, an alternating magnetic field with a nominal frequency of 183 kHz and a field amplitude of 17 kA m^−1^ was employed (fulfilling the conditions below the physiological limit for clinical applications), showing an increase in temperature up to 4 °C in the 5 mL samples containing a very low concentration (0.1 wt%) of the clusters under study. A much better performance was observed in the case of the clusters of Mn_0.6_Fe_2.4_O_4_ nanoparticles (blue curve in Fig. 7c). As mentioned in the case of the magnetic separation applications, the presence of Mn^2+^ cations being incorporated into the tetrahedral voids of the spinel structure implies an increased value of magnetization compared to magnetite. Consequently, a larger value of susceptibility at low fields increases the heat delivery related SAR value and explains the better result shown in Fig. 7c for the clusters of Mn_0.6_Fe_2.4_O_4_ nanoparticles. However, besides Mn doping, other parameters such as the size of the nanoparticles and the total size of the clusters are also key in the final heating performance,^51^ explaining the worse results for the clusters of Mn_0.3_Fe_2.7_O_4_ nanoparticles (red curve in Fig. 7c).

Another interesting approach to attain a high heat-delivery efficiency is to prepare anisotropic magnetic nanostructures, for example aligning Li_0.3_Zn_0.3_Co_0.1_Fe_2.3_O_4_ spinel ferrite nanoparticles onto multiwall carbon nanotubes.^76^ The magnetic particles were synthesized by a sol–gel method and then annealed at different temperatures, to be finally deposited onto carbon nanotubes. The Raman spectrum shows two differentiated regions, at low and high wavenumbers. For the former, nine bands associated with the lattice vibrations were visible. Among these, five of them correspond to the characteristic Raman modes of magnetite, that is, those centered at 254, 334, 466, 520 and 686 cm^−1^, related to the vibrational modes T_2g_(1), E_g_, T_2g_(2), T_2g_(3) and A_1g_, respectively. Additionally, four remaining bands stem from the partial filling of tetrahedral and octahedral positions by Li, Co, Zn and Fe ions, therefore splitting different vibrational modes. Finally, there is another vibrational feature centered at 288 cm^−1^, which matches that of the E_g_(2) mode of α-Fe_2_O_3_, and would confirm the presence of a secondary phase, likely due to the temperature at which calcination takes place (hematite is formed above 400 °C). However, there is no evidence of other oxidized phases, such as maghemite, since no additional modes appear at higher wavenumbers (*i.e.* 700 cm^−1^). The two bands at 1347 and 1576 cm^−1^ are attributed to the stretching modes of sp^2^ carbon stemming from the nanotube matrix. For the hyperthermia measurements, an alternating magnetic field working at a frequency of 300 kHz with a field amplitude of 33.4 kA m^−1^ was employed, showing an increase in temperature up to 42 °C within 5 minutes, for samples containing 2 mg mL^−1^ of the material. As mentioned in the previous case, the presence of small fractions of intercalating cations in the main structure can enhance the saturation magnetization,^77–79^ resulting in higher heating transmission. Thus, an analogous study was performed by the same group, incorporating Ni instead of Li, reporting an increase in temperature up to 42 °C within 10 minutes under similar working conditions.^80^

Another approach to obtain suitable structures for heat delivery was based on preparing anisotropic (elongated) nanomaterials by a chemical synthetic method. This is the case of Fe@Fe_2_O_3_ nanoworms synthesized by the reduction-assisted co-precipitation of iron sulfate.^81^ For that, individual metallic iron nanoparticles are formed as an initial stage, to subsequently and autonomously self-assemble into worms with the surface eventually oxidized. For the characterization of the sample, X-ray diffraction was used in order to corroborate the formation of the main phase (*i.e.* metallic iron). The additional peaks corresponding to the oxide were not clearly resolved, likely due to the lower intensities in contrast to the principal component, or because of having an amorphous and inhomogeneous shell. This is therefore a great example in which Raman spectroscopy emerges as a convenient tool to undoubtedly determine the nature of the oxide layer. The spectrum depicts the typical modes of hematite (α-Fe_2_O_3_), where five out of the seven permitted bands according to group theory, can be observed. Indeed, the bands from the sample are centered at 220, 286, 401, 495 and 599 cm^−1^, matching the A_1g_(1), E_g_(1), E_g_(4), A_1g_(2) and E_g_(5) vibrational modes.^38^ Note that cubic iron was not recorded by using Raman spectroscopy, since it does not present any vibrational mode that implies a change in the polarizability.

The heating ability of these samples was measured under different working conditions, obtaining the maximum efficiency for the most concentrated colloidal suspensions (10 mg mL^−1^), upon applying an alternating magnetic field at 75 kHz and 4.5 kA m^−1^. In this situation, the system reached a temperature of 92 °C after 10 minutes, and when the frequency was changed to 95 kHz, the same value of temperature was attained but just after ∼3 minutes. Since these extremely high temperatures might be not optimal for therapy purposes, reduced concentrations of the sample or different nominal frequencies were shown to provide sufficient energy to treat for example a malignant tissue.

### 4.3 Contrast agents for MRI

MRI is a technique based on nuclear magnetic resonance that provides different signals (contrast resolution) between neighboring regions, with a performance that can be increased by using contrast agents.^82^ It is based on the detection of short-time contrast differences, which the basic physical principle relates to the magnetic moment relaxation.

Basically, in order to obtain images with this technique, the region of interest is exposed to a primary magnetic field (*B*_0_), resulting in net magnetization (the majority of the hydrogen nuclear spins are aligned to this field in a lower-energy state and precess under the Larmor frequency) along the *z*-axis, that is, in the direction of the applied field. Subsequently, a radio-frequency pulse of a given amplitude perpendicular to this field is applied for a certain period of time, so that a large number of protons absorb energy and reach a higher-energy state, resulting in net magnetization in the *xy* plane. Once the radio-frequency excitation pulse stops, the net magnetization re-aligns along the *z*-axis, with the nuclear spins returning to the equilibrium state by a relaxation process.

The pursued contrast effect depends in fact on the longitudinal (related to the spin–lattice relaxation, *T*_1_) and transverse (related to the spin–spin relaxation, *T*_2_) relaxation times of the protons of the water present in the region to be imaged (80% *ca.* in living bodies), once exposed to the primary magnetic field.^83^ The *T*_1_ (or spin–lattice) relaxation time stems from the interactions of the spins with the surroundings. Thus, different tissues provide distinct MRI signals because of different interactions with the spins. Additionally, given the fact that every magnetic moment will be influenced by one another in close proximity, the relaxation process will be therefore different in the presence of magnetic nanoparticles. In such a situation, the spins of the protons will rotate at different and larger speeds once the pulse is removed, that is, not in a total *in-phase* situation but in a total increased *out-of-phase* situation, offering a larger *T*_2_ (or spin–spin) relaxation.

This *T*_2_ relaxation or *r*_2_ relaxivity is given by:
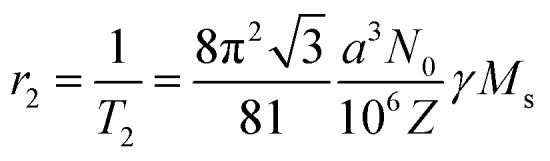
where *a* is the lattice parameter, *N*_0_ is the Avogadro constant, *Z* is the number of the formula units per unit cell, *γ* is the proton gyromagnetic ratio (42.6 MHz T^−1^), and *M*_S_ is the saturation magnetization.

The product *γM*_S_ represents the relaxation stemming from the field inhomogeneities causing spin–spin dephasing and providing the difference in contrast between distinct tissues. Nanoparticles with large values of saturation magnetization are therefore appropriate candidates to generate these field inhomogeneities when an external magnetic field is applied. In this manner, a thorough analysis of the magnetic nanoparticles to be used as contrast agents will be required, to clearly identify the iron oxide phases responsible of the increased value of saturation magnetization.

Therefore, while the small or large values of saturation magnetization of the nanoparticles to be used differentiate them already for MRI applications,^43,84^ Raman spectroscopy can shed light on the iron oxide phases responsible for these values. Accordingly, silica-coated magnetite–maghemite nanocrystals were likewise characterized, and though a noisy Raman spectrum rendered the assignation of the characteristic A_1g_ mode to the main vibrational modes of the magnetite or maghemite spinel lattices difficult, it pointed out the magnetite–maghemite oxidation transition. This characteristic is most likely responsible for the relatively low value of saturation magnetization measured.^43^ Alternatively, mesoporous silica particles that serve as the host for maghemite nanoparticles were employed, with the aim of developing superparamagnetic nanoarchitectures with high values of saturation magnetization and reduced Brownian motion.^85^ With this strategy, a 2× increase in the relaxivity value was reported, in contrast to free maghemite nanoparticles, and the absence of further oxidation of the maghemite phase was confirmed with Raman spectroscopy once it was included in the silica matrix.

## 5. Conclusions

This short review highlights the potential of Raman spectroscopy as a benchmark tool for the study of magnetic nanoparticles, showing unmatched capabilities for fast and easy characterization of complex multi-phase nanocomposites. This review emphasizes that while *a priori*, oxidation processes and phase transitions in nanoparticles imply in general a deterioration of the magnetic properties, the combination of different iron oxide phases in the same nanocrystal leads to a unique scenario in which different magnetic parameters can be tuned on demand. In this manner, a higher degree of control over the general magnetic properties of the material permits its easier implementation in important bio-related applications such as magnetic separation, hyperthermia and magnetic resonance imaging. Consequently, the combination of modern colloidal chemistry tools for the design of complex (multiphase) magnetic materials with structural analysis capabilities offered by Raman spectroscopy leads to an optimization of the magnetic performance needed. Such synergy can open the door to a completely new generation of advanced theranostic platforms.

## Conflicts of interest

There are no conflicts to declare.

## Supplementary Material

NA-001-C9NA00064J-s001

## References

[cit1] Laurent S., Forge D., Port M., Roch A., Robic C., Vander Elst L., Muller R. N. (2008). Chem. Rev..

[cit2] Yoo D.-W., Lee J.-H., Shin T.-H., Cheon J. (2011). Acc. Chem. Res..

[cit3] Lee J.-H., Kim J.-W., Cheon J. (2013). Mol. Cells.

[cit4] Colombo M., Carregal-Romero S., Casula M. F., Gutiérrez L., Morales M. P., Böhm I. B., Heverhagen J. T., Prosperi D., Parak W. J. (2012). Chem. Soc. Rev..

[cit5] Arami H., Khandhar A., Liggitt D., Krishnan K. M. (2015). Chem. Soc. Rev..

[cit6] Pankhurst Q. A., Connolly J., Jones S. K., Dobson J. (2003). J. Phys. D: Appl. Phys..

[cit7] Otero-Lorenzo R., Davila-Ibáñez A. B., Comesaña-Hermo M., Correa-Duarte M. A., Salgueiriño V. (2014). J. Mater. Chem. B.

[cit8] Fontaíña-Troitiño N., Rivas-Murias B., Rodríguez-González B., Salgueiriño V. (2014). Chem. Mater..

[cit9] Lee K., Lee S., Ahn B. (2019). Chem. Mater..

[cit10] Garnero C., Lepesant M., Marcelot C., Shin Y., Meny C., Farger P., Warot-Fonrose B., Arenal R., Viau G., Soulantica K., Fau P., Poveda P., Lacroix L.-M., Chaudret B. (2019). Nano Lett..

[cit11] Torruella P., Ruiz-Caridad A., Walls M., Roca A. G., López-Ortega A., Blanco-Portals J., López-Conesa L., Nogués J., Peiró F., Estradé S. (2018). Nano Lett..

[cit12] Slavov L., Abrashev M. V., Merodiiska T., Gelev Ch., Vandenberghe R. E., Markova-Deneva I., Nedkov I. (2010). J. Magn. Magn. Mater..

[cit13] Otero-Lorenzo R., Ramos-Docampo M. A., Rodríguez-González B., Comesaña-Hermo M., Salgueiriño V. (2017). Chem. Mater..

[cit14] Hosterman B. D., Farley J. W., Johnson A. L. (2013). J. Phys. Chem. Solids.

[cit15] Chandramohan P., Srinivasan M. P., Velmurugan S., Narasimhan S. V. (2011). J. Solid State Chem..

[cit16] Jubb A. M., Allen H. C. (2010). ACS Appl. Mater. Interfaces.

[cit17] Shebanova O. N., Lazor P. (2003). J. Raman Spectrosc..

[cit18] Rivas-Murias B., Salgueiriño V. (2017). J. Raman Spectrosc..

[cit19] Rodriguez R. D., Sheremet E., Deckert-Gaudig T., Chaneac C., Hietschold M., Deckert V., Zahn D. R. T. (2015). Nanoscale.

[cit20] Liu H. L., Su Y. C., Tang Y. H., Lin J. G. (2014). J. Appl. Phys..

[cit21] Shebanova O. N., Lazor P. (2003). J. Solid State Chem..

[cit22] de Faria D. L. A., Lopes F. N. (2007). Vib. Spectrosc..

[cit23] Testa-Anta M., Liébana-Viñas S., Rivas-Murias B., Rodríguez-González B., Farle M., Salgueiriño V. (2018). Nanoscale.

[cit24] Ferraro J. R. , Nakamoto K. and Brown C. W., Introductory Raman Spectroscopy, Elsevier, 2003

[cit25] Smith E. and Dent G., Modern Raman Spectroscopy. A Practical Approach, Chichester, Wiley, 2005

[cit26] Bernath P. F. , Spectra of Atoms and Molecules, Oxford University Press, Oxford, 2005

[cit27] Atkins P. W. , Child M. S. and Phillips C. S. G., Tables for Group Theory, Oxford University Press, Oxford, 2006

[cit28] White W. B., DeAngelis B. A. (1967). Spectrochim. Acta, Part A.

[cit29] Willock D. , Molecular Symmetry, Chichester Wiley, 2009

[cit30] Cornell R. M. and Schwertmann U., The Iron Oxides. Structure, Properties, Reactions, Occurrences and Uses, Wiley-VCH, Weinheim, 2003

[cit31] Hanesch M. (2009). Geophys. J. Int..

[cit32] Iliev M. N., Mazumdar D., Ma J. X., Gupta A., Rigato F., Fontcuberta J. (2011). Phys. Rev. B: Condens. Matter Mater. Phys..

[cit33] Verble J. L. (1974). Phys. Rev. B: Solid State.

[cit34] Laguna-Bercero M. A., Sanjuán M. L., Merino R. I. (2007). J. Phys.: Condens. Matter.

[cit35] Jørgensen J.-E., Mosegaard L., Thomsen L. E., Jensen T. R., Hanson J. C. (2007). J. Solid State Chem..

[cit36] Morales M. P., Pecharroman C., Gonzalez Carreño T., Serna C. J. (1994). J. Solid State Chem..

[cit37] Chamritski I., Burns G. J. (2005). J. Phys. Chem. B.

[cit38] de Faria D. L. A., Venâncio Silva S., De Oliveira M. T. (1997). J. Raman Spectrosc..

[cit39] Coey J. M. D. , Magnetism and Magnetic Materials, Cambridge University Press, New York, 2010

[cit40] Lee J. B., Kim H. J., Lužnik J., Jelen A., Pajić D., Wencka M., Jagličić Z., Meden A., Dolinšek J. (2014). J. Nanomater..

[cit41] Li S., Hihara L. H. (2015). J. Electrochem. Soc..

[cit42] Das S., Hendry M. J. (2011). Chem. Geol..

[cit43] Iqbal M. Z., Ma X., Zhang L., Ren W., Xiang L., Wu A. (2015). J. Mater. Chem. B.

[cit44] Aivazoglou E., Metaxa E., Hristoforou E. (2018). AIP Adv..

[cit45] Kertmen A., Torruella P., Coy E., Yate L., Nowaczyk G., Gapinski J., Vogt C., Toprak M., Estradé S., Peiró F., Milewski S., Jurga S., Andruzskiewicz R. (2017). Langmuir.

[cit46] Rikken R. S. M., Nolte R. J. M., Maan J. C., van Hest J. C. M., Wilson D. A., Christianen P. C. M. (2014). Soft Matter.

[cit47] Schattling P. S., Ramos-Docampo M. A., Salgueiriño V., Städler B. (2017). ACS Nano.

[cit48] Son S. J., Reichel J., He B., Schuchman M., Lee S. B. (2008). J. Am. Chem. Soc..

[cit49] Cao Z., Yue Z., Li X., Dai Z. (2013). Langmuir.

[cit50] Mhlanga N., Sinha Ray S., Lemmer Y., Wesley-Smith J. (2015). ACS Appl. Mater. Interfaces.

[cit51] McCloskey K. E., Chalmers J. J., Zborowski M. (2003). Anal. Chem..

[cit52] Liu F., KC P., Zhang G., Zhe J. (2016). Anal. Chem..

[cit53] Lim J., Lanni C., Evarts E. R., Lanni F., Tilton R. D., Majetich S. A. (2011). ACS Nano.

[cit54] Otero-Lorenzo R., Fantechi E., Sangregorio C., Salgueiriño V. (2016). Chem.–Eur. J..

[cit55] Ramos-Docampo M. A., Testa-Anta M., Rivas-Murias B., Salgueiriño V. (2019). J. Nanosci. Nanotechnol..

[cit56] Deng H., Li X., Peng Q., Wang X., Chen J., Li Y. (2005). Angew. Chem., Int. Ed..

[cit57] Yang P., Yuan X., Hu H., Liu Y., Zheng H., Yang D., Chen L., Cao M., Xu Y., Min Y., Li Y., Zhang Q. (2018). Adv. Funct. Mater..

[cit58] Zhang F., Zhao L., Wang S., Yang J., Lu G., Luo N., Gao X., Ma G., Xie H.-Y., Wei W. (2018). Adv. Funct. Mater..

[cit59] Wan L., Song H., Chen X., Zhang Y., Yue Q., Pan P., Su J., Elzatahry A. A., Deng Y. (2018). Adv. Mater..

[cit60] Wang Y., He J., Chen J., Ren L., Jiang B., Zhao J. (2012). ACS Appl. Mater. Interfaces.

[cit61] González-Domínguez E., Comesaña-Hermo M., Mariño-Fernández R., Rodríguez-González B., Arenal R., Salgueiriño V., Moldes D., Othman A. M., Pérez-Lorenzo M., Correa-Duarte M. A. (2016). ChemCatChem.

[cit62] Khurshid H., Li W., Chandra S., Phan M. H., Hadjipanayis G. C., Muherjee P., Srikanth H. (2013). Nanoscale.

[cit63] Pichon B. P., Gerber O., Lefevre C., Florea I., Fleutot S., Baaziz W., Pauly M., Ohlmann M., Ulhaq C., Ersen O., Pierron-Bohnes V., Panissod P., Drillon M., Begin-Colin S. (2011). Chem. Mater..

[cit64] Estrader M., López-Ortega A., Golosovsky I. V., Estradé S., Roca A. G., Salazar-Álvarez G., López-Conesa L., Tobia D., Winkler E., Ardisson J. D., Macedo W. A. A., Morphis A., Vasilakaki M., Trohidou K. N., Gukasov A., Merebeau I., Makarova O. L., Zysler R. D., Peiró F., Baró M. D., Bergström L., Nogués J. (2015). Nanoscale.

[cit65] Lak A., Niculaes D., Anyfantis G. C., Bertoni G., Barthel M. J., Marras S., Cassani M., Nitti S., Athanassiou A., Giannini C., Pellegrino T. (2016). Sci. Rep..

[cit66] Sun X., Huls N. F., Sigdel A., Sun S. (2012). Nano Lett..

[cit67] Simeonidis K., Mourdikoudis S., Tsiaoussis I., Rangis N., Angelakeris M., Kalogirou O., Delimitis A., Dendrinou-Samaria C. (2007). Mod. Phys. Lett. B.

[cit68] Pereira C., Pereira A. M., Fernandez C., Rocha M., Menes R., Fernández-García M. P., Guedes A., Tavares P. B., Greneche J.-M., Araújo J. P., Freire C. (2012). Chem. Mater..

[cit69] Nguyen X. S., Zhang G., Yang X. (2017). ACS Appl. Mater. Interfaces.

[cit70] Hergt R., Dutz S. (2007). J. Magn. Magn. Mater..

[cit71] Shliomis M. I. and Stepanov V. I., in Relaxation Phenomena in Condensed Matter, ed. W. T. Coffey, Wiley, New York, 1994

[cit72] Lima Jr E., de Biasi E., Zyslar R. D., Vasquez M. M., M-Pisciotti M. L., Torres T. E., Calatayud M. P., Marquina C., Ibarra M. R., Goya G. F. (2014). J. Nanopart. Res..

[cit73] Périgo E. A., Hemery G., Sandre O., Ortega D., Garaio E., Plazaola F., Terán F. J. (2015). Appl. Phys. Rev..

[cit74] Rosensweig E. R. (2002). J. Magn. Magn. Mater..

[cit75] Julien C. M., Massot M., Poinsignon C. (2004). Spectrochim. Acta, Part A.

[cit76] Dalal M., Greneche J.-M., Satpati B., Ghzaiel T. B., Mazaleyrat F., Ningthoujam R. S., Chakrabarti P. K. (2017). ACS Appl. Mater. Interfaces.

[cit77] Gorter E. V. (1950). Nature.

[cit78] Bercoff P. G., Bertorello H. R. (2000). J. Magn. Magn. Mater..

[cit79] Fontaíña-Troitiño N., Ramos-Docampo M. A., Testa-Anta M., Rodríguez-González B., Bañobre-López M., Bocher L., McKenna K. P., Salgueiriño V. (2018). J. Mater. Chem. C.

[cit80] Dalal M., Das A., Das D., Ningthoujam R. S., Chakrabarti P. K. (2018). J. Magn. Magn. Mater..

[cit81] Wang L., Lu X., Wang J., Yang S., Song X. (2016). J. Alloys Compd..

[cit82] Na H. B., Song I. C., Hyeon T. (2009). Adv. Mater..

[cit83] Brown M. A. and Smelka R. C., MRI: Basic principles and applications, Wiley, New York, 2003

[cit84] Lee N., Choi Y., Lee Y., Park M., Moon W. K., Choi S. H., Hyeon T. (2012). Nano Lett..

[cit85] Saikia K., Bhattacharya K., Sen D., Kaushik S. D., Biswas J., Lodha S., Gogoi B., Buragohain A. K., Kockenberger W., Deb P. (2019). Appl. Surf. Sci..

