# Supplementary material for: Raman spectroscopy to unravel the magnetic properties of iron oxide nanocrystals for bio-related applications
Source: Nanoscale Adv. 2019 Apr 23;1(6):2086–103. doi: 10.1039/c9na00064j (PMC9418671; doi:10.1039/c9na00064j)
Supplement: NA-001-C9NA00064J-s001 [file NA-001-C9NA00064J-s001.pdf]

Electronic Supplementary Material (ESI) for Nanoscale Advances.
This journal is © The Royal Society of Chemistry 2019

Electronic Supporting Information

Raman Spectroscopy to unravel Magnetic Properties of Iron Oxide Nanocrystals for Bio-related Applications

Martín Testa-Anta, Miguel A. Ramos-Docampo, Miguel Comesaña-Hermo,

Beatriz Rivas-Murias, Verónica Salgueiriño *

Departamento de Física Aplicada, Universidade de Vigo, 36310 Vigo, Spain.

Université Paris Diderot, Sorbonne Paris Cité, ITODYS, UMR CNRS 7086, 75013, Paris, France

1. Description of the Raman equipment

Figure S1 illustrates schematically the main parts of a Raman spectrometer. The sample (a monocrystal or powder of nanocrystals) is placed on a glass slide and illuminated by a monochromatic light source (laser) through a microscope objective. According to the scheme, this microscope objective collects both the incident and the scattered light and regulates as well the laser power illuminating the sample. To detect the small fraction of the Raman scattered radiation, the elastically-scattered light is first cut out by a filter, which is often a holographic notch or dielectric edge filter, such that the Raman scattered light can be subsequently focused by different optics and then split into its component wavelengths using a grating monochromator. At that point, a charge-coupled device (CCD) is used for detecting the intensity of the Raman scattered light.

Eventually, this signal is transferred to a computer where it is processed, displayed and stored by means of a specific software, which also controls the instrument and its motors.

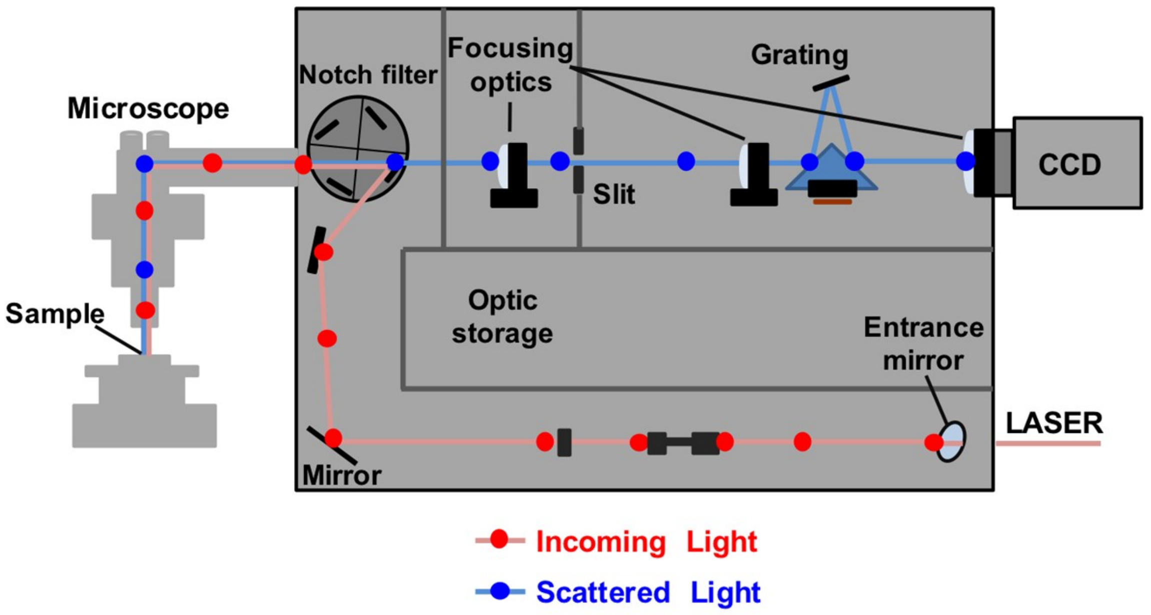

Figure S1. Scheme of a Raman spectrometer and microscope (using a visible laser) showing the path for the incoming (red) and scattered (blue) light.

For a Raman spectrum to be accurately registered, the adjustment of the laser excitation wavelength is crucial,[1] given the fact that it determines the excitation efficiency, the fluorescence, and the heat absorption of the sample. Since the Raman scattering efficiency is proportional to λ^{-4} (see equation 7 in the article, showing the Raman scattering intensity to be proportional to the fourth power of the incident light frequency (ν_0)), the scan time becomes reduced as the laser wavelength decreases, while keeping constant all other conditions. Nevertheless, fluorescence emission processes may also interfere with the Raman scattering in the final spectra. Since the

fluorescence interference occurs at a lower energy than that of the excitation frequency, longer excitation wavelengths will give rise to better results in this regard.[2]

Furthermore, heat absorption is also an important process that can take place while registering a Raman spectrum, especially in the case of dark samples. In general, when using longer excitation wavelengths more light is absorbed and more heat is transferred to the sample, offering therefore broader Raman modes that can appear shifted to lower frequencies. Over a threshold, the transferred heat can modify the sample under study, inducing for example oxidation processes or structural transitions,[3-5] or even damaging the sample completely.

Typical commercial lasers used for Raman spectroscopy provide the following wavelengths: 532 nm (frequency doubled Nd:YAG and Nd:YVO_4 diode), 633 nm (He-Ne laser), 785 nm (Near Infrared (NIR) diode laser), 830 nm (GaAlAs) and 1064 nm (Nd:$Y_3Al_5O_{12}$).

The spectra registered using different excitation wavelength can present slightly different features stemming from the following linked factors: a) the optical confocal depth, that decreases with decreasing wavelength for a given material, b) absorption, since a decrease in the laser wavelength can lead to an increase in absorption and consequent reduction of the penetration depth, and c) Raman cross section, the Raman intensity of a given material, depending on the exciting wavelength can lead to resonant Raman effects.[6]

2. Application of Group Theory to a discrete molecule AB_4

Aiming to clarify how group theory can be applied so as to figure out the optical phonon modes that will be Raman active for a particular crystal lattice, the theoretical concepts explained in Section 2.2 are herein detailed step-by-step. For simplicity, and owing to the large number of atoms present within a typical spinel unit cell, a discrete molecule has been selected to this end. Namely,

a tetrahedral molecule AB_4 (for example, methane (CH_4) or carbon tetrachloride (CCl_4)) will be used to determine the symmetry of the corresponding Raman active modes.

A general procedure to carry out this task involves the following steps:

i) **Determine the point group of the molecule under consideration and its corresponding character table**. Assuming a perfectly symmetric tetrahedral AB_4 molecule (see Figure S2a), the system belongs to the point group T_d. The corresponding character table is shown in Table S-I.[7]

Table S-I. Character table for the T_d point group.

T_d ($\bar{4}3m$)	E	$8C_3$	$3C_2$	$6S_4$	$6\sigma_d$		(h=24)
A_1	1	1	1	1	1		$x^2+y^2+z^2$
A_2	1	1	1	-1	-1		
E	2	-1	2	0	0		$(2z^2-x^2-y^2, x^2-y^2)$
T_1	3	0	-1	1	-1	(R_x,R_y,R_z)	
T_2	3	0	-1	-1	1	(x,y,z)	(xy,xz,yz)

ii) **Choose a vector basis v to describe the molecule, and determine a reducible representation of the system in terms of that basis**. A simple vector basis v to describe the system can be defined by the three coordinate axes on each atom comprising the molecule (as schematically shown in Figure S2b).

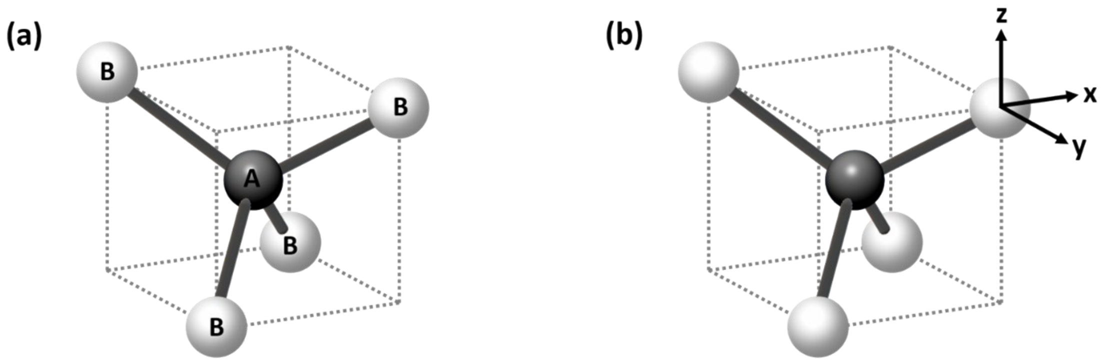

Figure S2. Scheme of a discrete AB_4 molecule belonging to the T_d point group (a) and of the Cartesian coordinates used as vector basis to describe the atomic displacements (b).

A reducible representation in terms of the basis v can be determined by analyzing the effect of each symmetry operation R within the point group on the coordinate axes of each atom, according to the following equation (eq. 12 in Section 2.2):

$$\chi(R) = \omega(R)(\pm 1 + 2cos\theta) \qquad (eq. S1)$$

where $\chi(R)$ is the character of the reducible representation for the symmetry operation R, $\omega(R)$ the number of atoms that remain invariant under the same operation and θ the rotation angle.

1) Identity operation, E

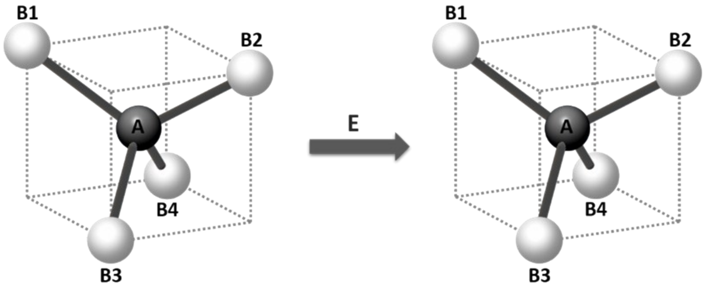

Figure S3. Scheme of the identity operation, E.

- Number of invariant/unshifted atoms: 5 (atoms A, B1, B2, B3 and B4).
- The identity operation can be regarded as a 0º proper rotation. Then:

$$\chi(E) = \omega(E)(1 + 2cos\theta) = 5 \cdot (1 + 2\cos(0°)) = 15$$

2) Three-fold proper rotation, C_3

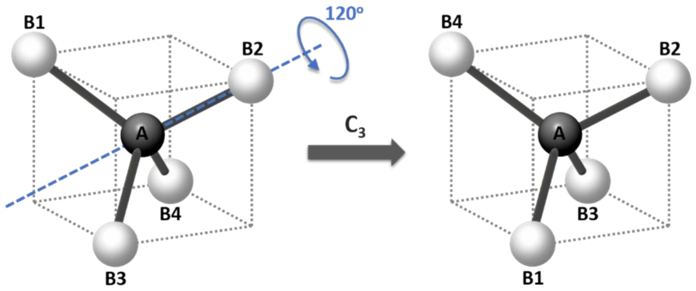

Figure S4. Scheme of the three-fold proper rotation, C_3.

- Number of invariant/unshifted atoms: 2 (atoms A and B2).
- A three-fold proper rotation implies a 120º rotation angle. Then:

$$\chi(C_3) = \omega(C_3)(1 + 2cos\theta) = 2 \cdot (1 + 2\cos(120°)) = 0$$

3) Two-fold proper rotation, C_2

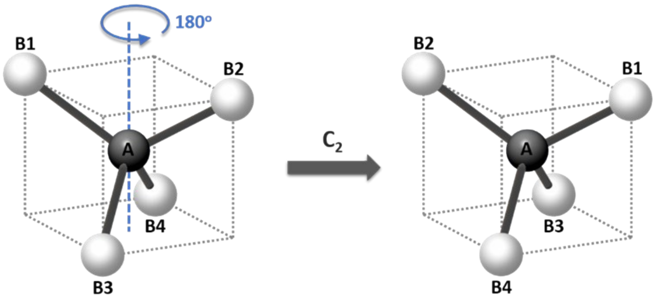

Figure S5. Scheme of the two-fold proper rotation, C_2.

- Number of invariant/unshifted atoms: 1 (atom A).
- A two-fold proper rotation implies a 180º rotation angle. Then:

$$\chi(C_2) = \omega(C_2)(1 + 2cos\theta) = 1 \cdot (1 + 2\cos{(180°)}) =- 1$$

4) Four-fold improper rotation, S_4

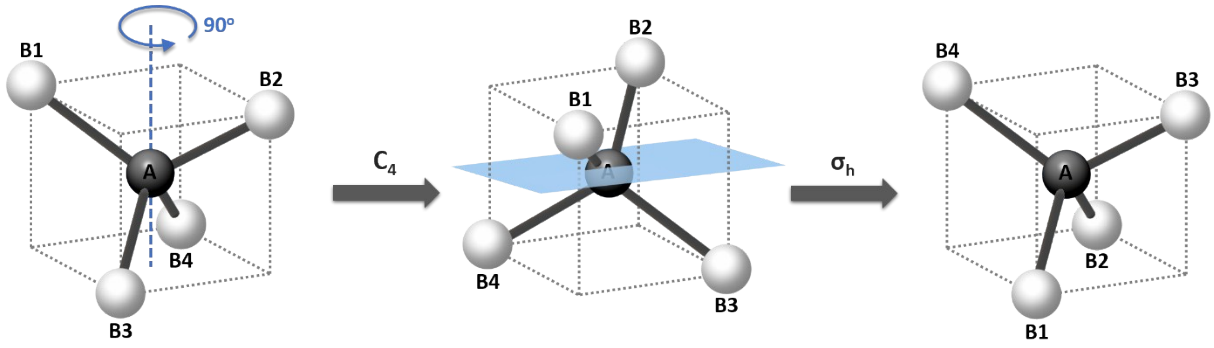

Figure S6. Scheme of the four-fold improper rotation, S_4.

- Number of invariant/unshifted atoms: 1 (atom A).
- A four-fold improper rotation implies a 90º improper rotation angle. Then:

$$\chi(S_4) = \omega(S_4)(- 1 + 2cos\theta) = 1 \cdot (- 1 + 2\cos{(90°)}) =- 1$$

5) Diagonal/dihedral reflection plane, σ_d

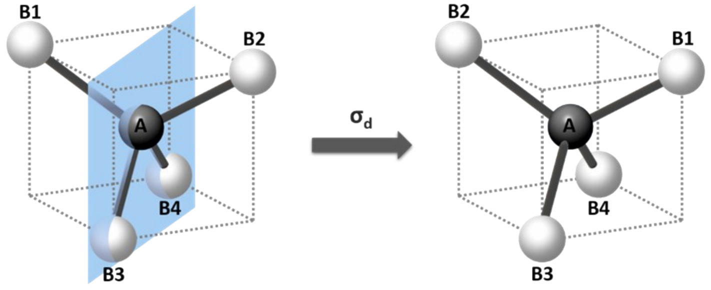

Figure S7. Scheme of the diagonal/dihedral reflection plane, σ_d.

- Number of invariant/unshifted atoms: 3 (atoms A, B3 and B4).
- A diagonal reflection plane can be regarded as a 0º improper rotation. Then:

  $\chi(\sigma_d) = \omega(\sigma_d)(-1 + 2cos\theta) = 3 \cdot (-1 + 2\cos(0°)) = 3$

The as-obtained reducible representation is summarized in Table S-II.

Table S-II. Reducible representation of the AB_4 molecule.

	E	$8C_3$	$3C_2$	$6S_4$	$6\sigma_d$
ω	5	2	1	1	3
Γ_{3N}	15	0	-1	-1	3

iii) **Apply the reduction formula in order to express the reducible representation in terms of the irreducible representations contained in the point group**. The reducible representation, Γ_{3N}, can be now expressed as a linear combination of the irreducible representations, $\Gamma_i^{(i)}$ (included in the T_d point group), applying the reduction formula (eq. 13-14 in Section 2.2):

$$\Gamma_{3N} = \sum_i a_i \Gamma_i^{(i)} \quad ; \quad a_i = \frac{1}{h}\sum_R \chi(R) \cdot g(R) \cdot \chi_i^{(i)}(R) \qquad (eq. S2)$$

where $\chi(R)$ and $\chi_i^{(i)}(R)$ are the characters of the reducible and the i-th irreducible representation for the symmetry operation R respectively, $g(R)$ the number of symmetry operations R of the same class, and h the order of the group.

The order h of the group represents the total number of symmetry operations within the group, and can be readily calculated by taking into account the number of symmetry operations R of the same class included in the T_d point group:

$$h = \sum_R g(R) = g(E) + g(C_3) + g(C_2) + g(S_4) + g(\sigma_d) = 1 + 8 + 3 + 6 + 6 = 24$$

The calculation of the coefficients for each irreducible representation (according to eq. S2) is shown below:

$$a_{A_1} = \frac{1}{24}(15 \cdot 1 \cdot 1 + 0 \cdot 8 \cdot 1 + (-1) \cdot 3 \cdot 1 + (-1) \cdot 6 \cdot 1 + 3 \cdot 6 \cdot 1) = 1$$

$$a_{A_2} = \frac{1}{24}(15 \cdot 1 \cdot 1 + 0 \cdot 8 \cdot 1 + (-1) \cdot 3 \cdot 1 + (-1) \cdot 6 \cdot (-1) + 3 \cdot 6 \cdot (-1)) = 0$$

$$a_E = \frac{1}{24}(15 \cdot 1 \cdot 2 + 0 \cdot 8 \cdot (-1) + (-1) \cdot 3 \cdot 2 + (-1) \cdot 6 \cdot 0 + 3 \cdot 6 \cdot 0) = 1$$

$$a_{T_1} = \frac{1}{24}(15 \cdot 1 \cdot 3 + 0 \cdot 8 \cdot 0 + (-1) \cdot 3 \cdot (-1) + (-1) \cdot 6 \cdot 1 + 3 \cdot 6 \cdot (-1)) = 1$$

$$a_{T_2} = \frac{1}{24}(15 \cdot 1 \cdot 3 + 0 \cdot 8 \cdot 0 + (-1) \cdot 3 \cdot (-1) + (-1) \cdot 6 \cdot (-1) + 3 \cdot 6 \cdot 1) = 3$$

Therefore, the reducible representation can be reduced as follows:

$$\Gamma_{3N} = a_{A_1}A_1 + a_{A_2}A_2 + a_E E + a_{T_1}T_1 + a_{T_2}T_2 = A_1 + E + T_1 + 3T_2$$

iv) **Identify which of the previous irreducible representations correspond to molecular vibrations**. The previous expression constitutes the mechanical representation of the system, which includes the normal modes for all the molecular motions. Indeed, taking into account the degeneracy of each irreducible representation, the tetrahedral AB_4 molecule displays 15 degrees of freedom, in agreement with the 3N degrees of freedom expected for this system. Among them, three represent pure translations of the molecule, since all the atoms involved are displaced along the same direction at once, and are included under one T_2 mode. When considering a crystal lattice,

these normal modes are usually referred to as acoustic modes, since they propagate as sound waves through the crystal. They can be easily identified from the first order terms in the character table, which in the case of the T_d point group correspond to one triple-degenerate T_2 mode (see Table S-I). Additionally, for non-linear molecules three rotational degrees of freedom are also present. As it can be inferred from the character table, they are comprised under one T_1 mode.

By subtracting the translational ($\Gamma_{trans} = T_2$) and rotational ($\Gamma_{rot} = T_1$) modes from the reducible representation, we end up with the irreducible representations corresponding to the vibrational modes of the AB_4 molecule:

$$\Gamma_{vib} = \Gamma_{3N} - \Gamma_{trans} - \Gamma_{rot} = A_1 + E + 2T_2$$

For these vibrational modes to be Raman active a change in the polarizability is needed, whereas the selection rule for IR spectroscopy requires a change in dipolar moment. The quadratic terms in the character table lead to a change of the polarizability tensor, and hence determine the Raman active modes. These terms are associated to the A_1, E and T_2 irreducible representations, thereby rendering the four vibrational modes ($A_1 + E + 2T_2$) active in Raman spectroscopy. On the other hand, the terms displaying linear symmetry are the ones implying a change in dipole moment, meaning that only two vibrational modes ($2T_2$) will be detectable through IR spectroscopy.

Further insight into the nature of these vibrational modes can be gained reconsidering the theoretical analysis performed. In fact, instead of describing the atomic displacements (under each symmetry operation) in terms of the three coordinate axes on each atom, the use of internal coordinates allows to discern between the stretching and/or bending origin of these vibrational modes. These internal coordinates are based on the displacement of one atom with respect to another (generally the central atom) along the bond direction, as schematically shown in Figure S8.

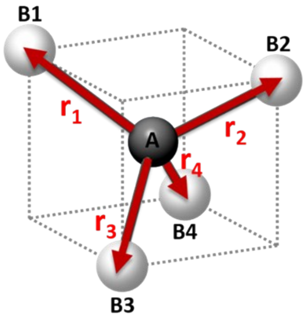

Figure S8. Scheme of the internal coordinates used as vector basis to describe the atomic displacements.

A reducible representation in terms of this new basis can be generated analyzing the effect of each symmetry operation R on the displacement vectors r_1, r_2, r_3 and r_4.

1) Identity operation, E

   - Number of invariant bonds: 4 (r_1, r_2, r_3 and r_4)

2) Three-fold proper rotation, C_3

   - Number of invariant bonds: 1 (r_2)

3) Two-fold proper rotation, C_2

   - Number of invariant bonds: 0

4) Four-fold improper rotation, S_4

   - Number of invariant bonds: 0

5) Diagonal/dihedral reflection plane, σ_d

   - Number of invariant bonds: 2 (r_3 and r_4)

Since the application of the symmetry operations does not modify the displacement vectors corresponding to the invariant bonds, the contribution of each symmetry operation (per invariant

bond) to the reducible representation is always 1. Therefore, the character of the reducible representation for the symmetry operation R is equal to the number of bonds that remain invariant under the same operation. Furthermore, bearing in mind the basis v considered, which was defined in terms of the relative displacement of each atom with respect to the central atom, the reducible representation does no longer represent the normal modes of all the molecular motions, but just the stretching vibrational modes of the AB_4 molecule. This reducible representation, denoted by $\Gamma_{stretching}$, is summarized in Table S-III.

Table S-III. Reducible representation of the AB_4 molecule as determined from the system internal coordinates.

	E	$8C_3$	$3C_2$	$6S_4$	$6\sigma_d$
Invariant bonds	4	1	0	0	2
$\Gamma_{stretching}$	4	1	0	0	2

The previous reducible representation can be expressed again in terms of the irreducible representations upon applying the reduction formula:

$$a_{A_1} = \frac{1}{24}(4 \cdot 1 \cdot 1 + 1 \cdot 8 \cdot 1 + 0 \cdot 3 \cdot 1 + 0 \cdot 6 \cdot 1 + 2 \cdot 6 \cdot 1) = 1$$

$$a_{A_2} = \frac{1}{24}(4 \cdot 1 \cdot 1 + 1 \cdot 8 \cdot 1 + 0 \cdot 3 \cdot 1 + 0 \cdot 6 \cdot (-1) + 2 \cdot 6 \cdot (-1)) = 0$$

$$a_E = \frac{1}{24}(4 \cdot 1 \cdot 2 + 1 \cdot 8 \cdot (-1) + 0 \cdot 3 \cdot 2 + 0 \cdot 6 \cdot 0 + 2 \cdot 6 \cdot 0) = 0$$

$$a_{T_1} = \frac{1}{24}(4 \cdot 1 \cdot 3 + 1 \cdot 8 \cdot 0 + 0 \cdot 3 \cdot (-1) + 0 \cdot 6 \cdot 1 + 2 \cdot 6 \cdot (-1)) = 0$$

$$a_{T_2} = \frac{1}{24}(4 \cdot 1 \cdot 3 + 1 \cdot 8 \cdot 0 + 0 \cdot 3 \cdot (-1) + 0 \cdot 6 \cdot (-1) + 2 \cdot 6 \cdot 1) = 1$$

$$\Gamma_{stretching} = a_{A_1}A_1 + a_{A_2}A_2 + a_E E + a_{T_1}T_1 + a_{T_2}T_2 = A_1 + T_2$$

The previous results indicate that, among the four vibrational modes of the AB_4 molecule, two of them ($A_1 + T_2$) correspond to stretching vibrations.

The bending vibrations can be easily determined by subtracting the stretching modes to the total number of vibrational modes:

$$\Gamma_{bending} = \Gamma_{vib} - \Gamma_{stretching} = E + T_2$$

Thus, the application of group theory to an AB_4 molecule belonging to the T_d point group predicts four Raman active ($A_1 + E + 2T_2$) and two IR active ($2T_2$) modes, typically labelled as $\upsilon_1(A_1)$, $\upsilon_2(E)$, $\upsilon_3(T_2)$ and $\upsilon_4(T_2)$.[6] Out of these vibrational modes, υ_1 and υ_3 correspond to purely stretching vibrations, whereas υ_2 and υ_4 involve bending vibrations, as shown in Figure S9.

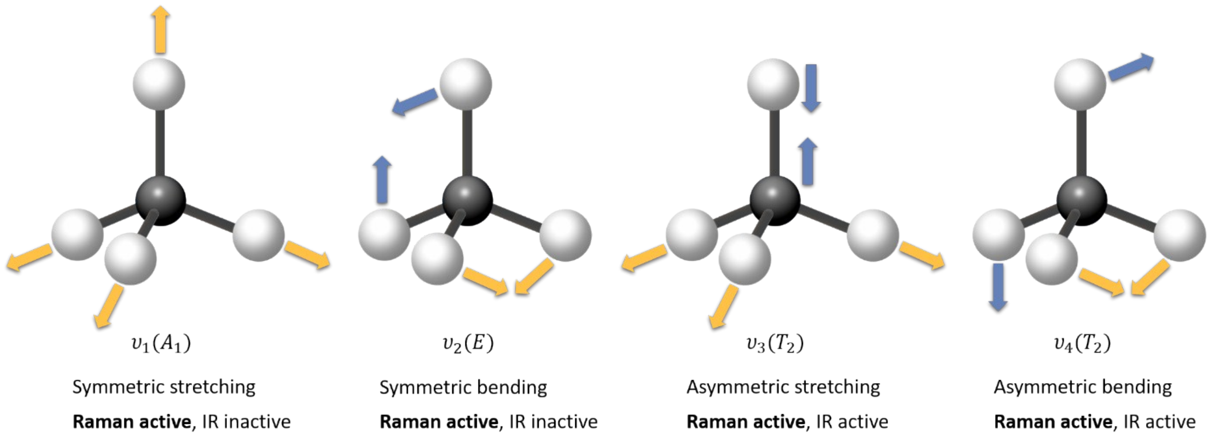

Figure S9. Scheme of the four vibrational modes of an AB_4 molecule belonging to the T_d point group.

3. Normal vibrations of the five Raman active modes in the cubic spinel structure

Considering a primitive unit cell of the spinel structure, which comprises 14 atoms and three individual units (two AO_4 units and one B_4 group) displaying tetrahedral symmetry (point group T_d), group theory predicts five Raman active modes, with schemes shown in Figure S10.

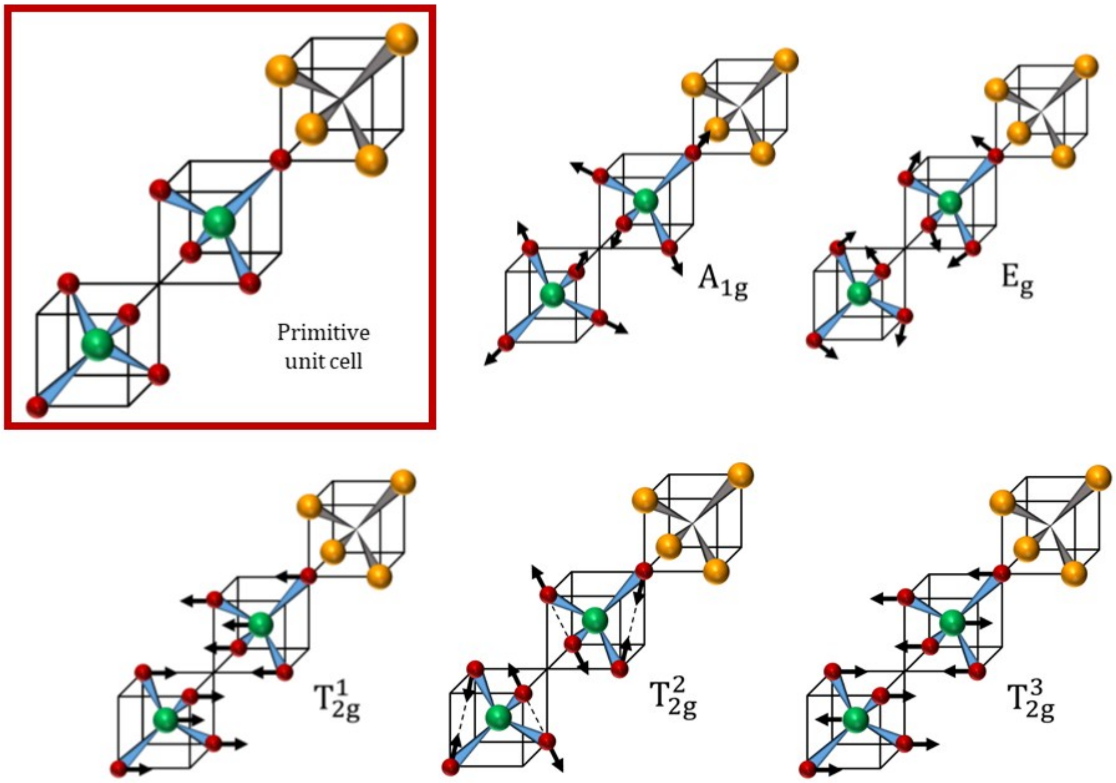

Figure S10. Schemes of the different vibration modes of the spinel structure (adapted from reference 8).

References

1. B&W Tek site, Application note: P. Zhou. Choosing the most Suitable Laser Wavelength for your Raman Application. http://bwtek.com/appnotes/choosing-the-most-suitable-laser-wavelength-for-your-raman-application (accessed January 2019).

2. E. Smith, G. Dent, *Modern Raman Spectroscopy. A Practical Approach*, Chichester, Wiley, 2005.

3. S. D. Harvey, T. J. Peters, B. W. Wright, *Appl. Spectrosc.* 2003, **57** (5), 580-587.

4. O. N. Shebanova, P. Lazor, *J. Raman Spectrosc.,* 2003, **34**, 845–852.

5. B. Rivas-Murias, V. Salgueiriño, *J. Raman Spectrosc.,* 2017, **48**, 837–841.

6. J. Kreisel, M. C. Weber, N. Dix, F. Sánchez, P. A. Thomas, J. Fontcuberta, *Adv. Func. Mater.,* 2012, **22**, 5044-5049.

7. P. W. Atkins, M. S. Child, C. S. G. Phillips, *Tables for Group Theory*. Oxford University Press, Oxford, 2006.

8. J. L. Verble, *Phys. Rev. B* 1974, **9**, 5236–5248.
